# Exploring the efficacy and beneficial population of preimplantation genetic testing for aneuploidy start from the oocyte retrieval cycle: a real-world study

**DOI:** 10.1186/s12967-023-04641-2

**Published:** 2023-11-02

**Authors:** Shujuan Ma, Jingnan Liao, Shuoping Zhang, Xiaoyi Yang, Berthold Hocher, Jing Tan, Yueqiu Tan, Liang Hu, Fei Gong, Pingyuan Xie, Ge Lin

**Affiliations:** 1 Clinical Research Center for Reproduction and Genetics in Hunan Province, Reproductive and Genetic Hospital of CITIC-Xiangya, No. 567, Tongzipo West Road, Yuelu District, Changsha, 410205 China; 2 NHC Key Laboratory of Human Stem Cell and Reproductive Engineering, Institute of Reproductive and Stem Cell Engineering, School of Basic Medical Science, Central South University, Changsha, China; 3 Fifth Department of Medicine, University Medical Centre Mannheim, University of Heidelberg, Mannheim, Germany; 4 Chinese Evidence-Based Medicine Center, West China Hospital, Sichuan University, Chengdu, China; 5 Hunan Normal University School of Medicine, Changsha, China; 6 National Engineering and Research Center of Human Stem Cells, Changsha, China

**Keywords:** Controlled ovarian stimulation, Cumulative live birth rate, In vitro fertilization, Neonatal malformation, Preimplantation genetic testing for aneuploidy

## Abstract

**Background:**

Preimplantation genetic testing for aneuploidy (PGT-A) is widely used as an embryo selection technique in in vitro fertilization (IVF), but its effectiveness and potential beneficiary populations are unclear.

**Methods:**

This retrospective cohort study included patients who underwent their first oocyte retrieval cycles at CITIC-Xiangya between January 2016 and November 2019, and the associated fresh and thawed embryo transfer cycles up to November 30, 2020. PGT-A (PGT-A group) and intracytoplasmic sperm injection (ICSI)/IVF (non-PGT-A group) cycles were included. The numbers of oocytes and embryos obtained were unrestricted. In total, 60,580 patients were enrolled, and baseline data were matched between groups using 1:3 propensity score matching. Sensitivity analyses, including propensity score stratification and traditional multivariate logistic regression, were performed on the original unmatched cohort to check the robustness of the overall results. Analyses were stratified by age, body mass index, ovarian reserve/responsiveness, and potential indications to explore benefits in subgroups. The primary outcome was cumulative live birth rate (CLBR). The other outcomes included live birth rate (LBR), pregnancy loss rate, clinical pregnancy rate, pregnancy complications, low birth weight rate, and neonatal malformation rate.

**Results:**

In total, 4195 PGT-A users were matched with 10,140 non-PGT-A users. A significant reduction in CLBR was observed in women using PGT-A (27.5% vs. 31.1%; odds ratio (OR) = 0.84, 95% confidence interval (CI) 0.78–0.91; *P* < 0.001). However, women using PGT-A had higher first-transfer pregnancy (63.9% vs. 46.9%; OR = 2.01, 95% CI 1.81–2.23; *P* < 0*.*001) and LBR (52.6% vs. 34.2%, OR = 2.13, 95% CI 1.92–2.36; *P* < 0*.*001) rates and lower rates of early miscarriage (12.8% vs. 20.2%; OR = 0.58, 95% CI 0.48–0.70; *P* < 0.001), preterm birth (8.6% vs 17.3%; *P* < 0.001), and low birth weight (4.9% vs. 19.3%; *P* < 0.001). Moreover, subgroup analyses revealed that women aged ≥ 38 years, diagnosed with recurrent pregnancy loss or intrauterine adhesions benefited from PGT-A, with a significant increase in first-transfer LBR without a decrease in CLBR.

**Conclusion:**

PGT-A does not increase and decrease CLBR per oocyte retrieval cycle; nonetheless, it is effective in infertile populations with specific indications. PGT-A reduces complications associated with multiple gestations.

**Supplementary Information:**

The online version contains supplementary material available at 10.1186/s12967-023-04641-2.

## Introduction

Embryo aneuploidy is the most common genetic abnormality in human blastocysts and is considered an important cause of low success rates during in vitro fertilization (IVF) [1]. Preimplantation genetic testing for aneuploidy (PGT-A) is used to identify euploid embryos prior to transfer to the uterus during IVF procedures. The first generation of PGT-A using fluorescence in situ hybridization was abandoned due to lack of efficacy [2, 3]. PGT-A based on comprehensive chromosomal screening is now widely used worldwide [4–6]. However, its effectiveness remains unclear [4, 7–13].

PGT-A is expensive; thus, identifying the patient population that would most benefit from this technique is imperative. According to the European Society of Human Reproduction and Embryology guidelines [14], the indications for PGT-A include advanced maternal age (AMA), recurrent pregnancy loss (RPL), recurrent implantation failure (RIF), and couples with severe male factor infertility. A multicenter, randomized control trial (RCT) revealed that a significant increase in ongoing pregnancy rate per embryo transfer was associated with PGT-A use in women aged 35–40 years with at least two embryos amenable to biopsy [9]. Another multicenter RCT confirmed that PGT-A neither increased live birth rates nor decreased miscarriage rates in patients with RPL and RIF [15]. However, several retrospective studies have suggested benefits of PGT-A, such as greater live birth rate (LBR) among women aged ≥ 35 years [16] and couples with RPL undergoing frozen-embryo transfer [17]. These conflicting results may be due to underlying selection bias toward good prognosis and normal ovarian reserve patients, while poor ovarian responders were still in question [9, 16, 18, 19]. Clinical outcomes were assessed based on transfer/detection cycles rather than oocyte retrieval cycles [9, 16], thereby not accounting for the effects of cycles without oocytes, absence of blastocysts for biopsy, or lack of euploid embryo for transfer on success rates. Moreover, some studies did not report cumulative success rates over time [9].

Therefore, in this retrospective cohort study, we compared single and cumulative transfer outcomes of first oocyte retrieval cycles in women with infertility with and without PGT-A after propensity score matching (PSM) to balance the clinical baseline. In addition, we examined the benefits derived from PGT-A across different age groups, body mass index (BMI), ovarian reserve/response, and potential indications via subgroup analyses.

## Materials and methods

### Study design and participants

This retrospective real-world study extracted patient information from the CITIC-Xiangya Assisted Reproductive Technology Cohort (NCT05404464). We included all registered patients who had their first oocyte retrieval cycle between January 1, 2016 and November 30, 2019, with the transfer date limited to November 30, 2020. For these women with infertility undergoing assisted reproduction for the first time, the indications for PGT-A in our center mainly included AMA (≥ 35 years), RPL (≥ 2 times), previous fetal malformations or chromosomal abnormalities, and severe male factors (severe oligospermia, asthenospermia, or teratospermia). The decision to perform PGT-A was made at the beginning of the cycle based on the indications and patient preference. Only PGT-A (PGT-A group) and intracytoplasmic sperm injection (ICSI)/IVF (non-PGT-A group) cycles were included. The Institutional Review Board of the Reproductive and Genetic Hospital of CITIC-Xiangya approved this study (LLSC2022034).

### ART treatments

Ovarian hyperstimulation protocols included long- and short-acting agonists, antagonists, and natural cycles, determined based on patients’ basal characteristics and performed as previously published [20]. For non-PGT-A cycles, embryos were cultured until the third day; in most cases, two cleavages were transferred according to morphological standards. When there were no high-quality D3 embryos (≥ 6C-II) or implantation failure, couples were advised to choose blastocyst culture until the fifth day, and no more than two blastocysts were transferred. Fresh transfers were canceled when patients were at high risk of ovarian hyperstimulation syndrome or poor endometrial receptivity, and embryos were vitrified and frozen for thawed transfer. For the PGT-A cycle, ICSI was performed on all Metaphase II oocytes. Both pronuclei embryos were cultured until day 5–6. Subsequently, approximately five trophoblast cells were biopsied. The blastocysts were then cryopreserved as previously described [21]. In most cases, a single euploid frozen-embryo transfer was performed each time. The endometrial preparation protocols for frozen transfer included the natural, hormone replacement (HRT), and GnRHa-HRT cycles. Luteal support was applied after dominant follicle ovulation in natural cycles, endometrial thickness ≥ 8 mm in HRT cycles, or oocyte retrieval in fresh transfers.

### PGT-A procedures

For PGT-A, the biopsy samples were subjected to DNA amplification using a PicoPLEX single-cell kit (Rubicon Genomics, Ann Arbor, MI, USA) according to the published protocol. The Illumina NextSeq platform (Illumina, San Diego, CA, USA)/BGI500 sequencer (BGI, Shenzhen, China) was used for next-generation sequencing, and at least 10 million single-end reads were obtained for each sample. The embryos were euploid, aneuploid, or mosaic (30–70%), as previously described [21, 22].

### Outcomes

The clinical outcomes were defined based on reporting guidelines [23, 24]. The primary outcome was the CLBR of the first oocyte retrieval. Only the first of multiple times of live births in an oocyte retrieval cycle was considered in the analysis. Secondary outcomes included LBR, clinical pregnancy rate (CPR), rate of pregnancy loss (ectopic pregnancy, early miscarriage, and late miscarriage), pregnancy complications (gestational hypertension and diabetes), preterm birth, low birth weight, neonatal malformations following the first transfer cycle, and cumulative singleton/multiple live birth and time to live birth (the interval between the delivery and oocyte retrieval dates). In addition, for each retrieval cycle, we compared the rates of cycles with oocyte non-retrieval, without transferable embryos, and with transferable embryos but without live birth. Transferable embryos were defined as those with cleavage stage > 6C-II or blastocyst stage > 4BC according to the Gardner criteria [25].

### Statistical analysis

PSM was used to balance the baseline characteristics and improve inter-group comparability [26]. Potential confounding factors were considered, including female age at the start of the cycle, male age (all ages were rounded down), male current smoking status, female BMI (kg/m^2^), anti-Mullerian hormone (AMH) levels, number of oocytes retrieved, and several indications for ART use (intrauterine adhesion, endometriosis, RPL, severe male factors). A 1:3 nearest-neighbor caliper matching method without replacement was used to match data between the PGT-A and non-PGT-A groups, using a caliper (0.02) of 0.2 of the standard deviation of the logit of the propensity score (0.1) [27, 28]. To check the overall results’ robustness, we conducted sensitivity analyses for the main outcomes, including propensity score stratification and traditional multivariable logistic regression in the original unmatched cohort [29].

Stratified analyses were performed according to female age at cycle start (five strata based on Society for Assisted Reproductive Technology (SART)-defined age groups: < 35, 35–38, 38–41,41–43, and ≥ 43, the ‘‘left close, right open’’ rule of thumb was applied), female BMI (three strata based on Chinese BMI classification [30]: < 18.5, 18.5–24, ≥ 24 kg/m^2^), ovarian reserve, and response (three strata based on the Poseidon criteria [31]: AFC < 5, AFC ≥ 5 and retrieved oocyte ≤ 9, AFC ≥ 5 and retrieved oocyte > 9), and potential indications (four strata: RPL, intrauterine adhesion, endometriosis, and severe male factor).

The most common indication for PGT-A in women < 35 years old was RPL; therefore, we further stratified this age group using the occurrence or absence of RPL. A 1:1 PSM was conducted in each stratum with the same matching parameters based on the overall analytic dataset to obtain the sub-analysis sets. The actual applied caliper values ranged from 0.01 to 0.03.

Data are presented as mean ± standard deviation, median with interquartile ranges (Q1–Q3), or numbers with percentages (%), as appropriate. A standardized mean difference (SMD) of < 0.2 indicated a negligible inter-group difference in the mean or covariate prevalence [28]. The normality of continuous variables was assessed using the Kolmogorov–Smirnov test. Normally- and non-normally-distributed continuous variables were analyzed using the two-sample t-test and Wilcoxon rank sum test, respectively. Pearson’s chi-squared or Fisher’s exact test was used for dichotomous variables. The odds ratio (OR) and 95% confidence interval (CI) were used to compare the outcome of interest between patients with and without PGT-A using logistic regression and displayed via forest plot. All statistical analyses were performed using R (version 4.2.1), and p-values < 0.05 were considered significant.

## Results

**Fig. 1:**
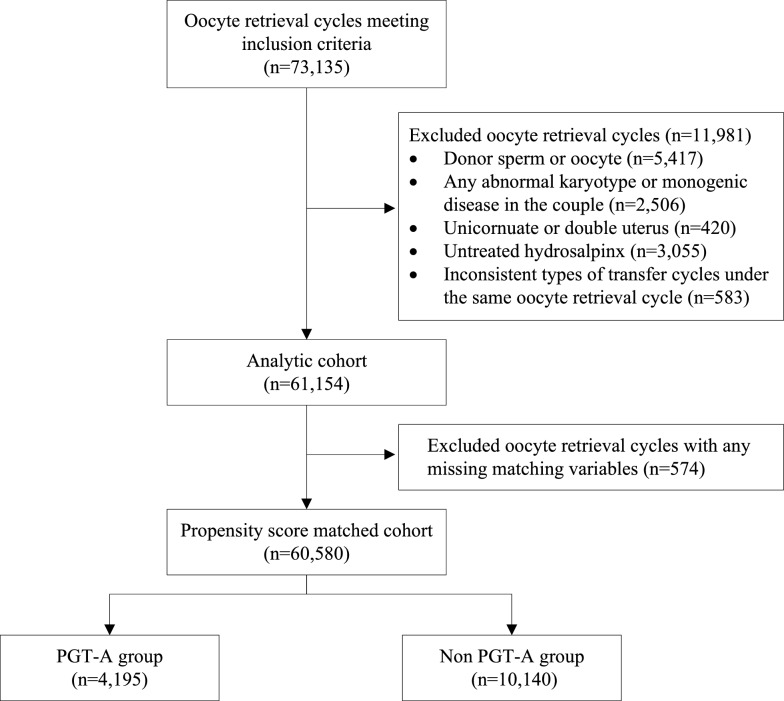
Study flowchart. PGT-A, preimplantation genetic testing for aneuploidy

Following the pre-designed inclusions and exclusions, 60,580 patients were included in the PSM for the overall cohort (Fig. 1), of which 4,409 (7.3%) underwent PGT-A. After 1:3 nearest-neighbor PSM matching, 4195 (95.1%) PGT-A users’ first oocyte retrieval cycles were matched to 10,140 oocyte retrieval cycles of non-users. Thus, the matching process resulted in a good balance for all covariates (SMD < 0.2) (Table 1 and Additional file 1: Figure S1).

**Table 1:** Demographic and clinical characteristics among unmatched and propensity score matched patients

	Unmatched			Matched		
	PGT-A (n = 4,409)	Non-PGT-A (n = 56,171)	SMD	PGT-A (n = 4195)	Non-PGT-A (n = 10,140)	SMD
Female age at cycle start, mean (± SD), years	38.16 (± 4.98)	31.64 (± 5.20)	1.310	38.13 (± 5.06)	38.49 (± 4.70)	− 0.177
Male age at cycle start, mean (± SD), years	40.23 (± 6.22)	33.83 (± 6.06)	1.028	40.18 (± 6.30)	40.60 (± 5.81)	− 0.138
Female BMI, mean (± SD), kg/m^2^	22.20 (± 2.32)	21.95 (± 2.66)	0.106	22.20 (± 2.33)	22.29 (± 2.38)	− 0.053
Basic AMH, mean (± SD), ng/ml	3.04 (± 3.22)	5.17 (± 4.54)	− 0.661	3.05 (± 3.24)	2.88 (± 3.10)	0.090
Number of oocytes retrieved, mean (SD)	8.06 (± 6.56)	10.84 (± 6.36)	− 0.425	8.05 (± 6.56)	7.73 (± 6.29)	0.090
Male current smoking, no. (%)			− 0.018			0.002
Yes	1345 (30.5)	18,153 (32.3)		1277 (30.4)	3027 (29.9)	
No	3064 (69.5)	38,018 (67.7)		2918 (69.6)	7113 (70.1)	
Intrauterine adhesion, no. (%)			0.006			0.003
Yes	600 (13.6)	7307 (13.0)		558 (13.3)	1169 (11.5)	
No	3809 (86.4)	48,864 (87.0)		3637 (86.7)	8971 (88.5)	
Endometriosis, no. (%)			− 0.007			0.005
Yes	391 (8.9)	5387 (9.6)		369 (8.8)	861 (8.5)	
No	4018 (91.1)	50,784 (90.4)		3826 (91.2)	9279 (91.5)	
RSA, no. (%)			0.239			0.060
Yes	1134 (25.7)	1002 (1.8)		924 (22.0)	954 (9.4)	
No	3275 (74.3)	55,169 (98.2)		3271 (78.0)	9186 (90.6)	
Severe male factor, no. (%)			− 0.025			− 0.001
Yes	575 (13.0)	8724 (15.5)		551 (13.1)	1364 (13.5)	
No	3834 (87.0)	47,447 (84.5)		3644 (86.9)	8776 (86.5)	

*AMH* antimullerian hormone, *BMI* body mass index, *Non-PGT-A* not use preimplantation genetic testing for aneuploidy, *PGT-A* preimplantation genetic testing for aneuploidy, *SMD* standardized mean difference, *RSA* recurrent spontaneous abortion

### Overall analyses

Women in the PGT-A (n = 4195) and non-PGT-A (n = 10,140) groups underwent 2258 and 9365 fresh- or thawed-embryo transfer cycles, yielding 1175 and 3818 newborns, respectively. Table 2 presents a comparison of outcomes post-PSM. The CLBR in the PGT-A group was significantly lower than that in the non-PGT-A group (27.5% vs. 31.1%; OR = 0.84; 95% CI 0.78–0.91; *P* < 0*.*001). However, the PGT-A group had a significantly higher LBR per transfer than the non-PGT-A group (51.2% vs. 33.6%; *P* < 0*.*001). Further analysis of cycles not achieving live birth revealed that due to the high proportion (23.0%) of detected abnormal embryos (including mosaic embryos), PGT-A users had a higher rate of cycles without euploid embryos (42.7% vs. 22.5%; *P* < 0*.*001). In addition, women not achieving live births in the PGT-A group were less likely to still have transferable embryos than those in the non-PGT-A group (10.1% vs. 16.5%; *P* < 0*.*001).

**Table 2:** Overall outcomes of the first and cumulative transplant between PGT-A and non-PGT-A patients

	PGT-A	Non-PGT-A	OR (95%CI)	P value
Number of oocyte retrieval cycles	4195	10,140		
Number of first transfer cycles	1844	7334		
Single embryo transfer	99.0% (1825/1844)	21.8% (1,599/7334)	344.50 (225.26–562.89)	< 0.001
Live birth rate	52.6% (970/1844)	34.2% (2,511/7334)	2.13 (1.92–2.36)	< 0.001
Singleton	52.0% (959/1844)	26.9% (1,975/7334)	2.94 (2.65–3.27)	< 0.001
Multiple	0.6% (11/1,844)	7.3% (536/7334)	0.08 (0.04–0.13)	< 0.001
Clinical pregnancy rate	63.9% (1,178/1844)	46.9% (3437/7334)	2.01 (1.81–2.23)	< 0.001
Singleton	62.5% (1,152/1844)	34.4% (2,522/7,334)	3.18 (2.86–3.53)	< 0.001
Multiple	1.4% (26/1844)	12.5% (915/7,334)	0.10 (0.07–0.15)	< 0.001
Pregnancy loss rate	17.7% (208/1178)	26.9% (926/3,437)	0.58 (0.49–0.69)	< 0.001
Ectopic pregnancy	0.9% (11/1178)	2.1% (71/3,437)	0.45 (0.22–0.81)	0.011
Early miscarriage	12.8% (151/1178)	20.2% (694/3,437)	0.58 (0.48–0.70)	< 0.001
Late miscarriage	4.0% (47/1178)	4.7% (161/3,437)	0.85 (0.60–1.17)	0.322
Pregnancy complications	33.3% (392/1178)	29.5% (1,014/3,437)	1.19 (1.03–1.37)	0.015
Gestational hypertension rate	4.1% (48/1178)	3.2% (111/3,437)	1.27 (0.89–1.79)	0.171
Gestational diabetes rate	17.4% (205/1178)	13.2% (453/3,437)	1.39 (1.16–1.66)	< 0.001
Preterm birth rate^a^	8.6% (83/970)	17.3% (432/2,502)	0.45 (0.35–0.57)	< 0.001
Singleton^a^	8.0% (77/959)	8.6% (169/1,970)	0.93 (0.70–1.23)	0.615
Multiple^a^	54.5% (6/11)	49.4% (263/532)	1.23 (0.37–4.30)	0.738
Low birth weight rate^a^	4.9% (48/972)	19.3% (579/3005)	0.22 (0.16–0.29)	< 0.001
Singleton^a^	4.0% (38/950)	4.8% (94/1950)	0.82 (0.55–1.20)	0.320
Multiple^a^	45.5% (10/22)	46.0% (485/1055)	0.98 (0.41–2.29)	0.962
Neonatal malformation cycle rate	0.9% (9/970)	2.0% (50/2511)	0.46 (0.21–0.90)	0.029
Cumulative number of transfer cycles	2258	9365		
Cumulative number of newborns	1175	3818		
Cumulative live birth rate^b^	27.5% (1155/4195)	31.1% (3150/10,140)	0.84 (0.78–0.91)	< 0.001
Singleton	27.2% (1141/4195)	24.7% (2508/10,140)	1.14 (1.05–1.23)	0.002
Multiple	0.3% (14/4195)	6.3% (642/10,140)	0.05 (0.03–0.08)	< 0.001
Interval since oocyte retrieval, days^a^	395 (348–473)	263 (254–347)	–	< 0.001
Number of cycles not reached live birth	3,040	6,990		
Oocyte unretrieved cycle rate	6.0% (251/4195)	6.4% (654/10,140)	0.92 (0.79–1.07)	0.296
No transferable embryo cycle rate^c^	42.7% (1793/4195)	22.5% (2281/10,140)	2.57 (2.38–2.78)	< 0.001
With transferable embryo cycle rate^d^	10.1% (423/4195)	16.5% (1671/10,140)	0.57 (0.51–0.64)	< 0.001

*CI* confidence interval, *Non-PGT-A* not use preimplantation genetic testing for aneuploidy, *OR* odds ratio, *PGT-A* preimplantation genetic testing for aneuploidy

^a^The missing conditions of these indicators in the PGT-A group and non-PGT-A group are as follows: 0/970 vs. 9/2511, 0/959 vs.5/1970, 0/11 vs.4/532, 9/981 vs. 45/3050, 9/959 vs. 25/1970, 0/22 vs. 20/1075, 9/1150 vs. 27/3143;

^b^Twice or more live births under the same oocyte retrieval cycle are counted as one when the cumulative live birth rate is calculated. The numbers of twice live births in the PGT-A group and non-PGT-A group were 6 and 23, respectively;

^c^The proportion of abnormal embryos (including chimeras) detected in the PGT-A group was 23.0% (965/4195);

^d^The undetected transferable embryos were included in the PGT-A group;

Comparisons around the first attempt suggested that women using PGT-A were more likely to achieve clinical pregnancy (63.9% vs. 46.9%; OR = 2.01, 95% CI 1.81–2.23; *P* < 0*.*001) and less likely to experience early miscarriage (12.8% vs. 20.2%; OR = 0.58; 95% CI 0.48–0.70; *P* < 0*.*001) than non-users. The chance of live birth resulting from the first transfer was significantly higher in the PGT-A than in the non-PGT-A group (52.6% vs. 34.2%; OR = 2.13; 95% CI 1.92–2.36; *P* < 0*.*001). A higher risk of gestational diabetes mellitus existed in the first PGT-A clinical pregnancy cycle (17.4% vs. 13.2%; *P* < 0*.*001). Meanwhile, a lower neonatal malformation risk (0.9% vs. 2.0%; *P* = 0.029) was observed in the first live birth cycle. Furthermore, 99.0% of PGT-A users had single blastocyst transfers; therefore, 78.2% of non-PGT-A users had double embryo transfers. Compared with PGT-A users, women in the IVF/ICSI group had significantly higher rates of multiple gestation (12.5% vs. 1.4%; *P* < 0*.*001), concomitant multiple live births (7.3% vs. 0.6%; *P* < 0*.*001), preterm birth (17.3% vs. 8.6%; *P* < 0*.*001), and low birth weight (19.3% vs. 4.9%; *P* < 0*.*001).

Our PSM method and two sensitivity analyses (Additional file 1: Figure S2) had similar results, suggesting that PGT-A use could significantly reduce the early miscarriage rate and increase the CPR and LBR for the first transfer cycle. However, the CLBR was significantly lower in women who underwent PGT-A than in those who did not.

### Subgroup analyses

**Fig. 2:**
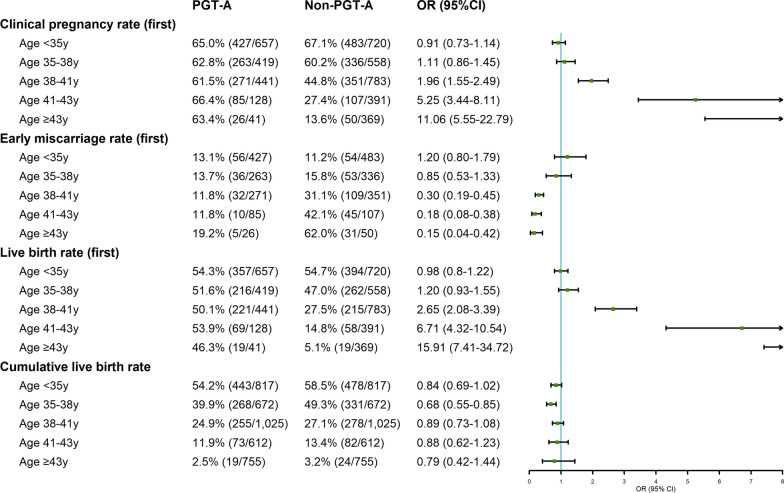
Forest plot for female age-stratified comparison of the primary outcomes between PGT-A and non-PGT-A groups. All ages were rounded down, and the “left close, right open” rule of thumb was applied uniformly for stratification variables. *CI* confidence interval; *OR* odds ratio, *PGT-A* preimplantation genetic testing for aneuploidy

Age-stratified analyses (Fig. 2 and Additional file 2: Table S1) suggested gradually increased differences in CPR, early miscarriage rate, and LBR between groups following the first transfer with increasing age strata. These indicators only differed significantly in women aged ≥ 38 years. Moreover, the CLBR of PGT-A users was lower than that of the control group in each age stratum, and the absolute difference in rates between the groups ranged from 0.7% to 9.4%. However, no significant difference existed in CLBR between groups in any age stratum, except for women aged 35–38 (39.9% vs. 49.3%; OR = 0.68; 95% CI 0.55–0.85; *P* < 0*.*001). In addition, among women aged ≥ 38 years, PGT-A users were less likely to still have transferable embryos than the controls (OR range: 0.05–0.39). Further stratified analysis (Additional file 2: Table S2) based on the occurrence or absence of RPL in women aged < 35 years revealed that PGT-A use significantly reduced the CLBR of the first oocyte retrieval cycle in young women without RPL (55.0% vs. 68.4%; OR = 0.56; 95% CI 0.41–0.78; *P* < 0*.*001). However, women aged < 35 years with RPL in the PGT-A group had a higher non-significant CLBR (54.0% vs. 51.7%; OR = 1.10; 95% CI 0.83–1.46; *P* = 0.517) than those in the non-PGT-A group.

In the BMI-stratified analyses (Additional file 2: Table S3 and Additional file 1: Figure S3), after a 1:1 PSM, only 166 women with a BMI < 18.5 in the PGT-A group were matched to those in the non-PGT-A group, and the absolute difference in CLBR between the groups was non-significant (33.1% vs. 38.0%; *P* = 0.359). Moreover, we observed no significant inter-group difference in early miscarriage rate (17.5% vs. 21.3%; *P* = 0.326) following the first transfer in sub-populations with BMI ≥ 24. Subgroup analyses based on ovarian reserve and response (Additional file 2: Table S4 and Additional file 1: Figure S4) revealed significant advantages of PGT-A for the CPR and LBR following the first transfer, and their negative impact on CLBR gradually decreased with improved ovarian function.

The analysis results stratified based on specific diseases are presented in Additional file 2: Tables S5 and Additional file 1: Figure S5. For women with RPL history, PGT-A significantly increased CPR (63.5% vs. 55.7%; OR = 1.39; 95% CI 1.08–1.78; *P* = 0.010) and LBR (51.5% vs. 42.3%; OR = 1.45; 95% CI 1.14–1.85; *P* = 0.003) in the first transfer cycle, without significantly reducing the early miscarriage rate (14.9 vs. 16.4%; OR = 0.89; 95% CI 0.58–1.38; *P* = 0.609) or CLBR (38.3% vs. 40.8%; OR = 0.90; 95% CI 0.73–1.11; *P* = 0.316). Similar results were observed in women with intrauterine adhesions. Meanwhile, in women with endometriosis, PGT-A use did not reduce the early miscarriage rate (22.0% vs. 20.4%, *P* = 0.809), but it reduced their CLBR (18.1% vs. 29.0%; OR = 0.54; 95% CI 0.36–0.80; *P* = 0.002). For couples with severe male fertility factors, although better pregnancy outcomes were observed in the PGT-A group following the first transfer, their CLBR (23.1% vs. 31.7%; OR = 0.65; 95% CI 0.49–0.86; *P* = 0.003) was significantly lower than that in the non-PGT-A group.

## Discussion

After balancing baseline characteristics using PSM, we observed that PGT-A does not increase CLBR. Women aged ≥ 38 years, diagnosed with RPL or intrauterine adhesions benefit more from PGT-A, reflected in significantly higher CPR and LBR following the first transfer, without reduced CLBR. Furthermore, the single blastocyst transfer in the PGT-A group reduced the complications associated with multiple gestations.

CLBR effectively evaluates PGT-A’s effectiveness, as it can comprehensively reflect the final treatment outcome of an oocyte retrieval cycle [32]. Our overall analysis, including all embryo transfers occurring ≥ 12 months after initial oocyte retrieval, suggested that PGT-A did not improve CLBR after balancing the clinical baseline. This finding was consistent with those of previous studies [15, 18], reflecting some PGT-A limitations. Studies had confirmed that some mosaic embryos might progress to healthy live births [33], and chromosomal mosaicism was a critical factor causing embryo wastage and reduced CLBR [34, 35]. The incidence of mosaic embryos in this study was 6.7% (603/8996), within the reported 2–13% range [36]. Actually, safety concerns had limited the transfer of mosaic embryos to only 50 patients, which had resulted in 19 successful live births. Of the 3040 PGT-A patients who did not achieve live births, 228 patients still had 271 mosaic embryos that were not selected for transfer. The majority of these patients opted for a new PGT-A cycle instead. Taking into account the potential for healthy deliveries from mosaic embryos, it is reasonable to estimate that CLBRs would increase to approximately 29.2–30.1% if all mosaic embryos were transferred [34, 35, 37]. Moreover, embryonic mosaicism also affected the accuracy of embryo biopsy for PGT-A, and trophectoderm results might not always represent whole-embryo genetic composition [38, 39]. For example, when segmental aneuploidy was identified using PGT-A, studies suggested that > 50% of segmental aneuploidies were derived from mitosis errors [21, 40]. Besides, the invasive biopsy during PGT-A might reduce embryo implantation potential [41, 42].

A healthy singleton live birth is the target outcome for all infertility treatments. However, multiple gestation is the most common complication of assisted reproductive treatment [43]. Multiple gestations were at significant risk for prematurity [44], hypertensive disorders [45], and neonatal and fetal demise [46]. PGT-A significantly reduced the ratio of preterm birth and low birth weight. These results were similar to those of previous studies [47].

AMA is an important indication for PGT-A as it increases the risk of meiotic chromosome segregation errors [7]. Various countries and reproductive centers define the AMA threshold differently, with most lying between 35 and 38 years of age. Age-stratified subgroup analysis suggested that women aged ≥ 38 years might benefit more from PGT-A treatment than those aged > 35 years. PGT-A use could decrease CLBR in women aged 35–37 years; however, CLBR did not differ significantly between groups in the ≥ 38 years age strata. This finding provides clinical evidence supporting PGT-A use in women aged ≥ 38 years.

Aneuploidy causes most early pregnancy losses; therefore, RPL is a suggested indication for PGT-A [17, 48]. However, little high-quality evidence supports PGT-A use in women with RPL [8]. Our results demonstrated significantly increased LBR and a non-significant decrease in early miscarriage rate in the first transfer cycle with PGT-A use in women with RPL. However, the CLBR exhibited no significant inter-group difference. In addition, high PGT-A usage among women < 35 years of age was due to RPL in our data. Therefore, we further stratified the analysis among these patients according to RPL diagnoses and observed that in young women with RPL, PGT-A omission improved LBR in the first transfer cycle and achieved higher CLBR (without significance). Our findings should encourage clinicians to discuss PGT-A use in patients with RPL.

Finally, our results revealed that severe male infertility factors may not be an appropriate indication for PGT-A. Poor sperm quality may be associated with lower fertilization rate and embryo development potential but not with the euploidy rate [49, 50]. Origin analysis of whole chromosome aneuploidies in blastomeres and blastocysts suggested that 80–90% of chromosome aneuploidies affect maternally-derived chromosomes [51, 52]. Several retrospective analyses indicated a significantly higher rate of mosaic blastocysts in the male/severe male factor infertility groups than in the non-male factor infertility group [53–55]. However, the majority of mosaic embryos were not transferred to the uterus. These may potentially explain why patients with severe male infertility factors derived minimal benefits from aneuploidy screening. Instead, they experienced embryo wastage as a result of PGT-A, leading to a significant reduction in CLBR.

Future studies are required to increase the extent of PGT-A embryo utilization, particularly in improving the accuracy of diagnosing mosaicism and elucidating the clinical significance of chromosome mosaicism, which will improve embryo selection and clinical management.

This retrospective analysis had several advantages over previous studies. First, using the complete data chain, we considered the full impact of oocyte retrieval or blastocyst formation on the comparison, starting with the patients’ wishes at the start of the cycle. Second, we analyzed CLBR based on the oocyte retrieval cycle rather than the transfer/detection cycle. Third, we used real-world data to reduce the selection bias caused by inappropriate inclusion. Nonetheless, this study has certain limitations. First, its retrospective design introduces inevitable bias. Second, some transferable embryos were not transferred in cycles that did not achieve live birth, especially in PGT-A cycles, of which 10.1% retained euploid embryos. We included the rate of these cycles in the two-group comparison; however, it could not fully reflect the true LBR. Third, these data were derived from a single IVF center; therefore, our results’ generalizability may be limited. Thus, multicenter studies with large data volumes are required to further confirm these findings.

## Conclusions

Regardless of the mechanism responsible, this large real-world database study verified that PGT-A does not increase and might even decrease CLBR per oocyte retrieval cycle. However, PGT-A is undeniably effective in patients with specific indications. Careful selection of suitable populations and appropriate clinical management of mosaic embryos are important for effective PGT-A implementation.

### Supplementary Information

**Additional file 1: Figure S1**. Distribution of distance among women who did (1) and did not (0) use preimplantation genetic testing for aneuploidy before and after the propensity score matching.** Figure S2**. Forest plot for sensitivity analyses of the primary outcomes between PGT-A and non-PGT-A groups. CI, confidence interval; OR, odds ratio; PGT-A, preimplantation genetic testing for aneuploidy. PSM, propensity score matching; PS stratification, propensity score stratification.** Figure S3**. Forest plot for female BMI-stratified comparison of the primary outcomes between PGT-A and non-PGT-A groups. BMI, body mass index; CI, confidence interval; OR, odds ratio; PGT-A, preimplantation genetic testing for aneuploidy.** Figure S4**. Forest plot for ovarian reserve and response-stratified comparison of the primary outcomes between PGT-A and non-PGT-A groups. AFC, antral follicle count; CI, confidence interval; OR, odds ratio; PGT-A, preimplantation genetic testing for aneuploidy.** Figure S5.** Forest plot for specific indication-stratified comparison of the primary outcomes between PGT-A and non-PGT-A groups. CI, confidence interval; OR, odds ratio; RPL, recurrent pregnancy loss; PGT-A, preimplantation genetic testing for aneuploidy.

**Additional file 2: Table S1.** Female age-stratified outcomes of the first and cumulative transplant between PGT-A and non-PGT-A patients. **Table S2.** Stratified analyses of the first and cumulative transplant between PGT-A and non-PGT-A group by the presence or absence of RPL in women aged <35 years. **Table S3.** Female BMI-stratified outcomes of the first and cumulative transplant between PGT-A and non-PGT-A patients. **Table S4.** Ovarian reserve and response-stratified outcomes of the first and cumulative transplant between PGT-A and non-PGT patients. **Table S5.** Specific indication-stratified outcomes of the first and cumulative transplant between PGT-A and non-PGT-A patients.

## Data Availability

The datasets used and analyzed during the current study are available from the corresponding author upon reasonable request.

## Abbreviations

PGT-A: Preimplantation genetic testing for aneuploidy
IVF: In vitro fertilization
CLBR: Cumulative live birth rate
LBR: Live birth rate
CPR: Clinical pregnancy rate
AMA: Advanced maternal age
RPL: Recurrent pregnancy loss
RIF: Recurrent implantation failure
RCT: Randomized control trial
PSM: Propensity score matching
OR: Odds ratio
CI: Confidence interval

## References

[1] Gleicher N, Orvieto R (2017). Is the hypothesis of preimplantation genetic screening (PGS) still supportable? A review. J Ovarian Res.

[2] Fritz MA (2008). Perspectives on the efficacy and indications for preimplantation genetic screening: where are we now?. Hum Reprod.

[3] Mastenbroek S, Twisk M, van Echten-Arends J (2007). In vitro fertilization with preimplantation genetic screening. N Engl J Med.

[4] Gleicher N, Patrizio P, Brivanlou A (2021). preimplantation genetic testing for aneuploidy—a castle built on sand. Trends Mol Med.

[5] Fiorentino F, Bono S, Biricik A (2014). Application of next-generation sequencing technology for comprehensive aneuploidy screening of blastocysts in clinical preimplantation genetic screening cycles. Hum Reprod.

[6] Yin X, Tan K, Vajta G (2013). Massively parallel sequencing for chromosomal abnormality testing in trophectoderm cells of human blastocysts. Biol Reprod.

[7] Rubio C, Bellver J, Rodrigo L (2017). In vitro fertilization with preimplantation genetic diagnosis for aneuploidies in advanced maternal age: a randomized, controlled study. Fertil Steril.

[8] Practice Committees of the American Society for Reproductive Medicine and the Society for Assisted Reproductive Technology (2018). Electronic address: practice committees of the american society for reproductive medicine and the society for assisted reproductive technology the use of preimplantation genetic testing for aneuploidy (PGT-A): a committee opinion. Fertil Steril.

[9] Munné S, Kaplan B, Frattarelli JL (2019). Preimplantation genetic testing for aneuploidy versus morphology as selection criteria for single frozen-thawed embryo transfer in good-prognosis patients: a multicenter randomized clinical trial. Fertil Steril.

[10] Roberts SA, Wilkinson J, Vail A, Brison DR (2022). Does PGT-A improve assisted reproduction treatment success rates: what can the UK register data tell us?. J Assist Reprod Genet.

[11] Cheng X, Zhang Y, Deng H (2022). Preimplantation genetic testing for aneuploidy with comprehensive chromosome screening in patients undergoing in vitro fertilization: a systematic review and meta-analysis. Obstet Gynecol.

[12] Simopoulou M, Sfakianoudis K, Maziotis E (2021). PGT-A: who and when? Α systematic review and network meta-analysis of RCTs. J Assist Reprod Genet.

[13] Dahdouh EM (2021). Preimplantation genetic testing for aneuploidy: a review of the evidence. Obstet Gynecol.

[14] Carvalho F, Coonen E, ESHRE PGT Consortium Steering Committee (2020). ESHRE PGT consortium good practice recommendations for the organisation of PGT. Hum Reprod Open.

[15] Sato T, Sugiura-Ogasawara M, Ozawa F (2019). Preimplantation genetic testing for aneuploidy: a comparison of live birth rates in patients with recurrent pregnancy loss due to embryonic aneuploidy or recurrent implantation failure. Hum Reprod.

[16] Haviland MJ, Murphy LA, Modest AM (2020). Comparison of pregnancy outcomes following preimplantation genetic testing for aneuploidy using a matched propensity score design. Hum Reprod.

[17] Bhatt SJ, Marchetto NM, Roy J, Morelli SS, McGovern PG (2021). Pregnancy outcomes following in vitro fertilization frozen embryo transfer (IVF-FET) with or without preimplantation genetic testing for aneuploidy (PGT-A) in women with recurrent pregnancy loss (RPL): a SART-CORS study. Hum Reprod.

[18] Yan J, Qin Y, Zhao H (2021). Live Birth with or without preimplantation genetic testing for aneuploidy. N Engl J Med.

[19] Lee E, Illingworth P, Wilton L, Chambers GM (2015). The clinical effectiveness of preimplantation genetic diagnosis for aneuploidy in all 24 chromosomes (PGD-A): systematic review. Hum Reprod.

[20] Li Y, Li X, Yang X (2019). Cumulative live birth rates in low prognosis patients according to the POSEIDON criteria: an analysis of 26,697 cycles of in vitro fertilization/intracytoplasmic sperm injection. Front Endocrinol.

[21] Xie P, Liu P, Zhang S (2022). Segmental aneuploidies with 1 Mb resolution in human preimplantation blastocysts. Genet Med.

[22] Zhou S, Xie P, Zhang S (2021). Complex mosaic blastocysts after preimplantation genetic testing: prevalence and outcomes after re-biopsy and re-vitrification. Reprod Biomed Online.

[23] Duffy JMN, Bhattacharya S, Bhattacharya S (2021). Standardizing definitions and reporting guidelines for the infertility core outcome set: an international consensus development study. Fertil Steril.

[24] Sun Q, Huang G, Sun H (2018). CSRM consensus on key indicators for quality control in IVF laboratory. J Reprod Med.

[25] ESHRE Special Interest Group of Embryology and Alpha Scientists in Reproductive Medicine (2017). Electronic address: The Vienna consensus: report of an expert meeting on the development of ART laboratory performance indicators. Reprod Biomed Online.

[26] Garrido MM, Kelley AS, Paris J (2014). Methods for constructing and assessing propensity scores. Health Serv Res.

[27] Caliendo M, Kopeinig S (2008). Some practical guidance for the implementation of propensity score matching. J Econom Surv.

[28] Abadie A, Imbens G (2016). Matching on the estimated propensity score. Econometrica.

[29] Austin PC (2011). An Introduction to propensity score methods for reducing the effects of confounding in observational studies. Multivariate Behav Res.

[30] WHO Expert Consultation (2004). Appropriate body-mass index for Asian populations and its implications for policy and intervention strategies. Lancet.

[31] Alviggi C, Andersen CY, Buehler K (2016). A new more detailed stratification of low responders to ovarian stimulation: from a poor ovarian response to a low prognosis concept. Fertil Steril.

[32] Harbin Consensus Conference Workshop Group (2014). Improving the reporting of clinical trials of infertility treatments (IMPRINT): modifying the CONSORT statement. Fertil Steril.

[33] Zhang YX, Chen JJ, Nabu S (2020). The pregnancy outcome of mosaic embryo transfer: a prospective multicenter study and meta-analysis. Genes.

[34] Capalbo A, Poli M, Rienzi L (2021). Mosaic human preimplantation embryos and their developmental potential in a prospective, non-selection clinical trial. Am J Hum Genet.

[35] Viotti M, Victor AR, Barnes FL (2021). Using outcome data from one thousand mosaic embryo transfers to formulate an embryo ranking system for clinical use. Fertil Steril.

[36] Popovic M, Dhaenens L, Boel A, Menten B, Heindryckx B (2020). Chromosomal mosaicism in human blastocysts: the ultimate diagnostic dilemma. Hum Reprod Update.

[37] Scott RT, de Ziegler D, Pirtea P, Jalas C (2022). Limits imposed by the experimental design of a large prospective non-inferiority study on PGT-A invalidate many of the conclusions. Hum Reprod.

[38] Victor AR, Griffin DK, Brake AJ (2019). Assessment of aneuploidy concordance between clinical trophectoderm biopsy and blastocyst. Hum Reprod.

[39] Popovic M, Dheedene A, Christodoulou C (2018). Chromosomal mosaicism in human blastocysts: the ultimate challenge of preimplantation genetic testing?. Hum Reprod.

[40] Girardi L, Serdarogullari M, Patassini C (2020). Incidence, origin, and predictive model for the detection and clinical management of segmental aneuploidies in human embryos. Am J Hum Genet.

[41] Zhang S, Luo K, Cheng D (2016). Number of biopsied trophectoderm cells is likely to affect the implantation potential of blastocysts with poor trophectoderm quality. Fertil Steril.

[42] Neal SA, Franasiak JM, Forman EJ (2017). High relative deoxyribonucleic acid content of trophectoderm biopsy adversely affects pregnancy outcomes. Fertil Steril.

[43] Hermans FJR, Schuit E, Bekker MN (2018). Cervical pessary after arrested preterm labor: a randomized controlled trial. Obstet Gynecol.

[44] Martin JA, Hamilton BE, Osterman MJK, Driscoll AK (2019). Births: final data for 2018. Natl Vital Stat Rep.

[45] Sibai BM, Hauth J, Caritis S (2000). Hypertensive disorders in twin versus singleton gestations national institute of child health and human development network of maternal-fetal medicine units. Am J Obstet Gynecol.

[46] Scher AI, Petterson B, Blair E (2002). The risk of mortality or cerebral palsy in twins: a collaborative population-based study. Pediatr Res.

[47] Yang Z, Liu J, Collins GS (2012). Selection of single blastocysts for fresh transfer via standard morphology assessment alone and with array CGH for good prognosis IVF patients: results from a randomized pilot study. Mol Cytogenet.

[48] Coonen E, Rubio C, ESHRE PGT-SR/PGT-A Working Group (2020). ESHRE PGT Consortium good practice recommendations for the detection of structural and numerical chromosomal aberrations. Hum Reprod Open.

[49] Mazzilli R, Cimadomo D, Vaiarelli A (2017). Effect of the male factor on the clinical outcome of intracytoplasmic sperm injection combined with preimplantation aneuploidy testing: observational longitudinal cohort study of 1219 consecutive cycles. Fertil Steril.

[50] Polese R, Scarselli F, Dale B, Minasi MG, Greco E (2022). Can sperm quality influence embryo development and its ploidy? Analysis of 811 blastocysts obtained from different sperm sources. Zygote.

[51] Kubicek D, Hornak M, Horak J (2019). Incidence and origin of meiotic whole and segmental chromosomal aneuploidies detected by karyomapping. Reprod Biomed Online.

[52] Tšuiko O, Vanneste M, Melotte C (2021). Haplotyping-based preimplantation genetic testing reveals parent-of-origin specific mechanisms of aneuploidy formation. NPJ Genom Med.

[53] Tarozzi N, Nadalini M, Lagalla C, Coticchio G, Zacà C, Borini A (2019). Male factor infertility impacts the rate of mosaic blastocysts in cycles of preimplantation genetic testing for aneuploidy. J Assist Reprod Genet.

[54] Kahraman S, Sahin Y, Yelke H (2020). High rates of aneuploidy, mosaicism and abnormal morphokinetic development in cases with low sperm concentration. J Assist Reprod Genet.

[55] Rodrigo L, Clemente-Císcar M, Campos-Galindo I, Peinado V, Simón C, Rubio C (2020). Characteristics of the IVF cycle that contribute to the incidence of mosaicism. Genes.

