# Supplementary figures and images for: Exploring the efficacy and beneficial population of preimplantation genetic testing for aneuploidy start from the oocyte retrieval cycle: a real-world study
Source: J Transl Med. 2023 Nov 2;21:779. doi: 10.1186/s12967-023-04641-2 (PMC10623718; doi:10.1186/s12967-023-04641-2)

Figure S1

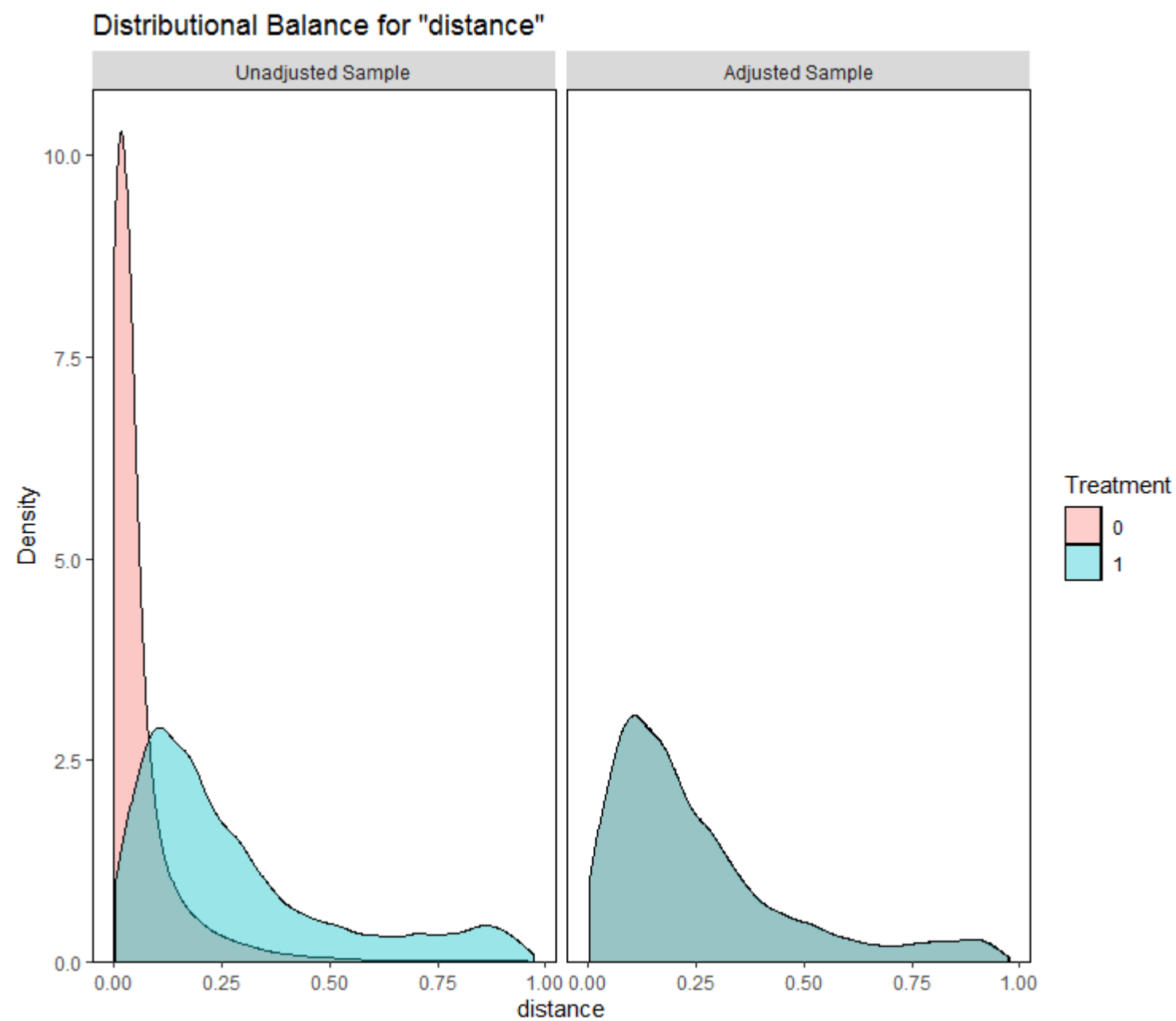

Figure S2

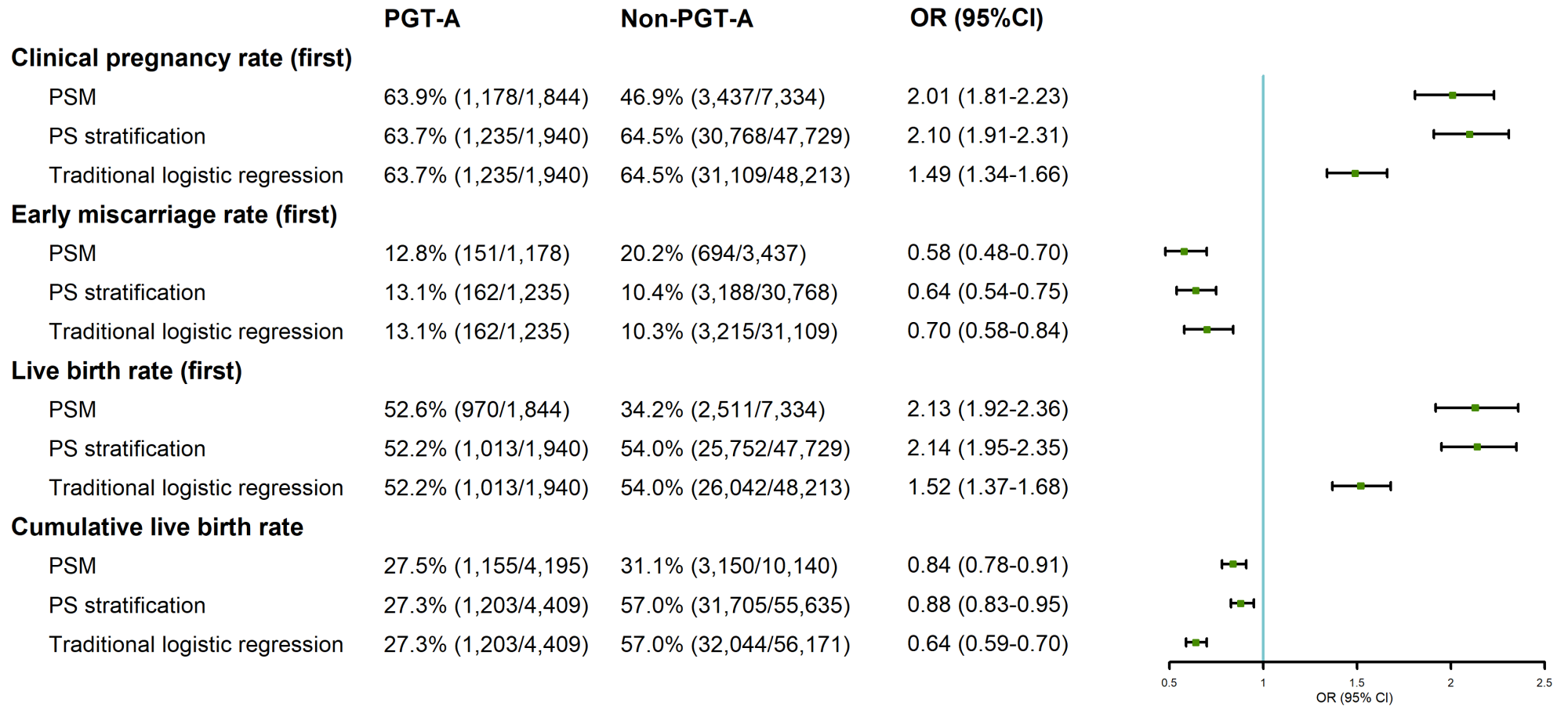

Figure S3

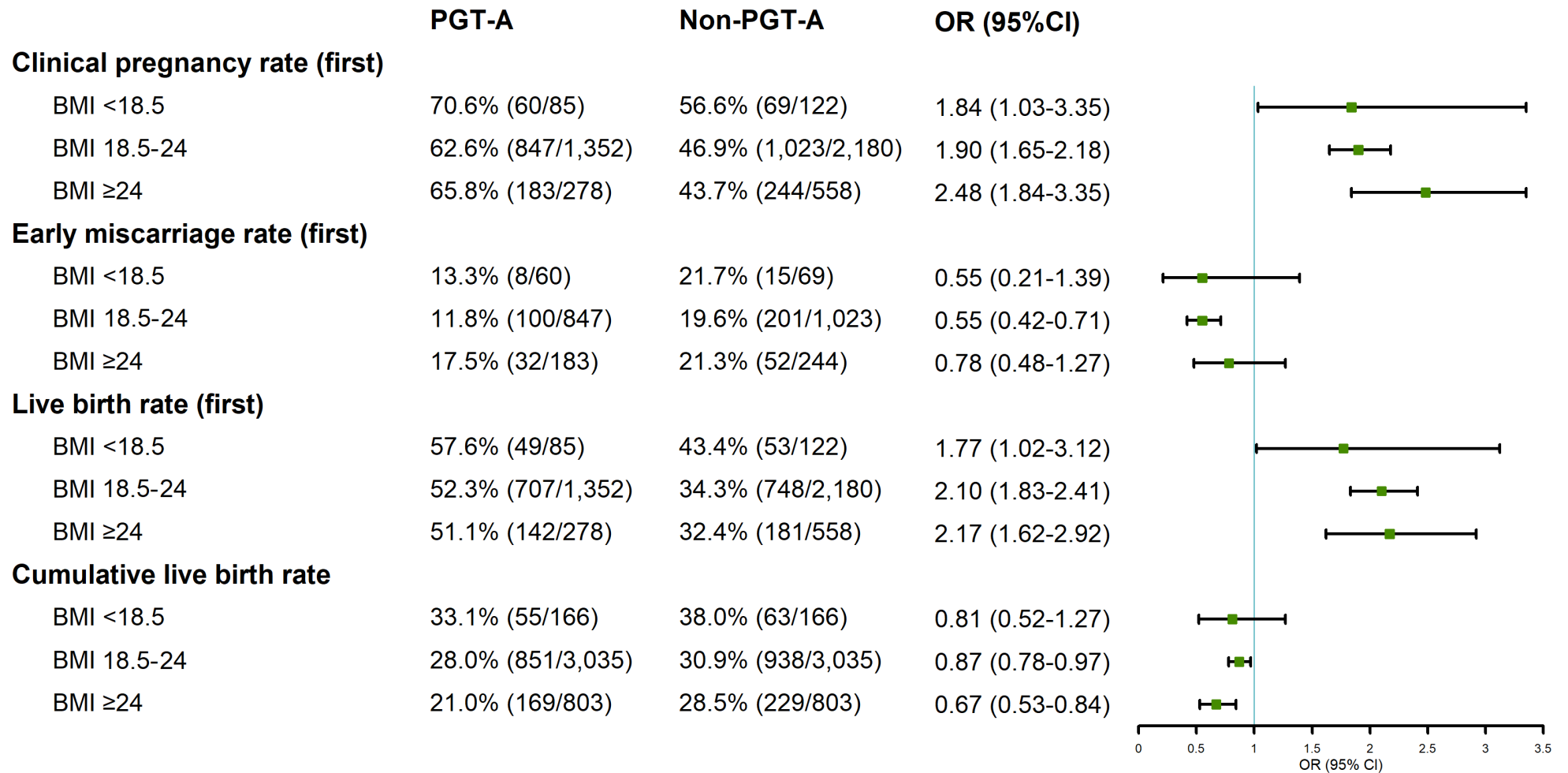

Figure S4

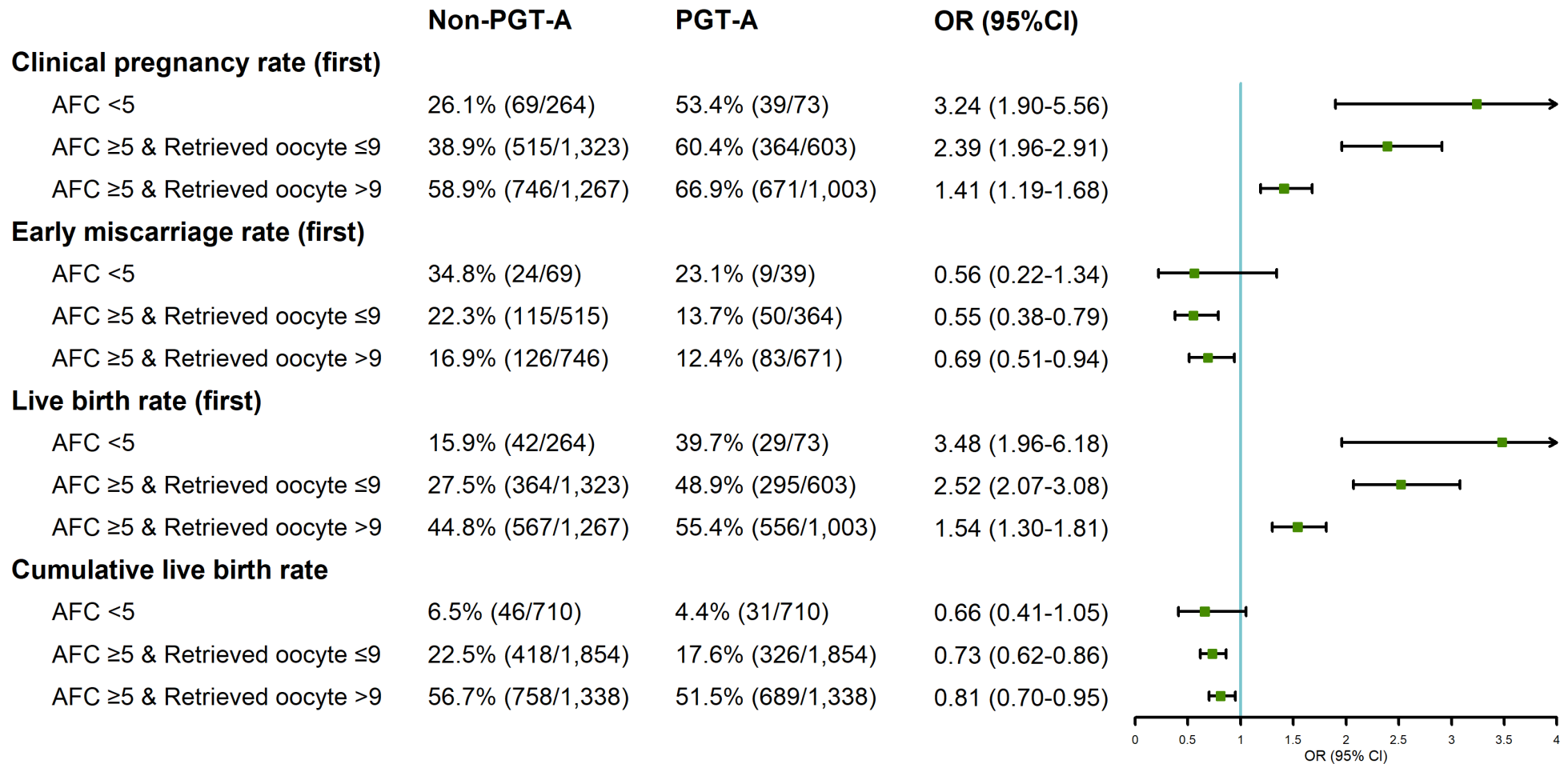

Figure S5

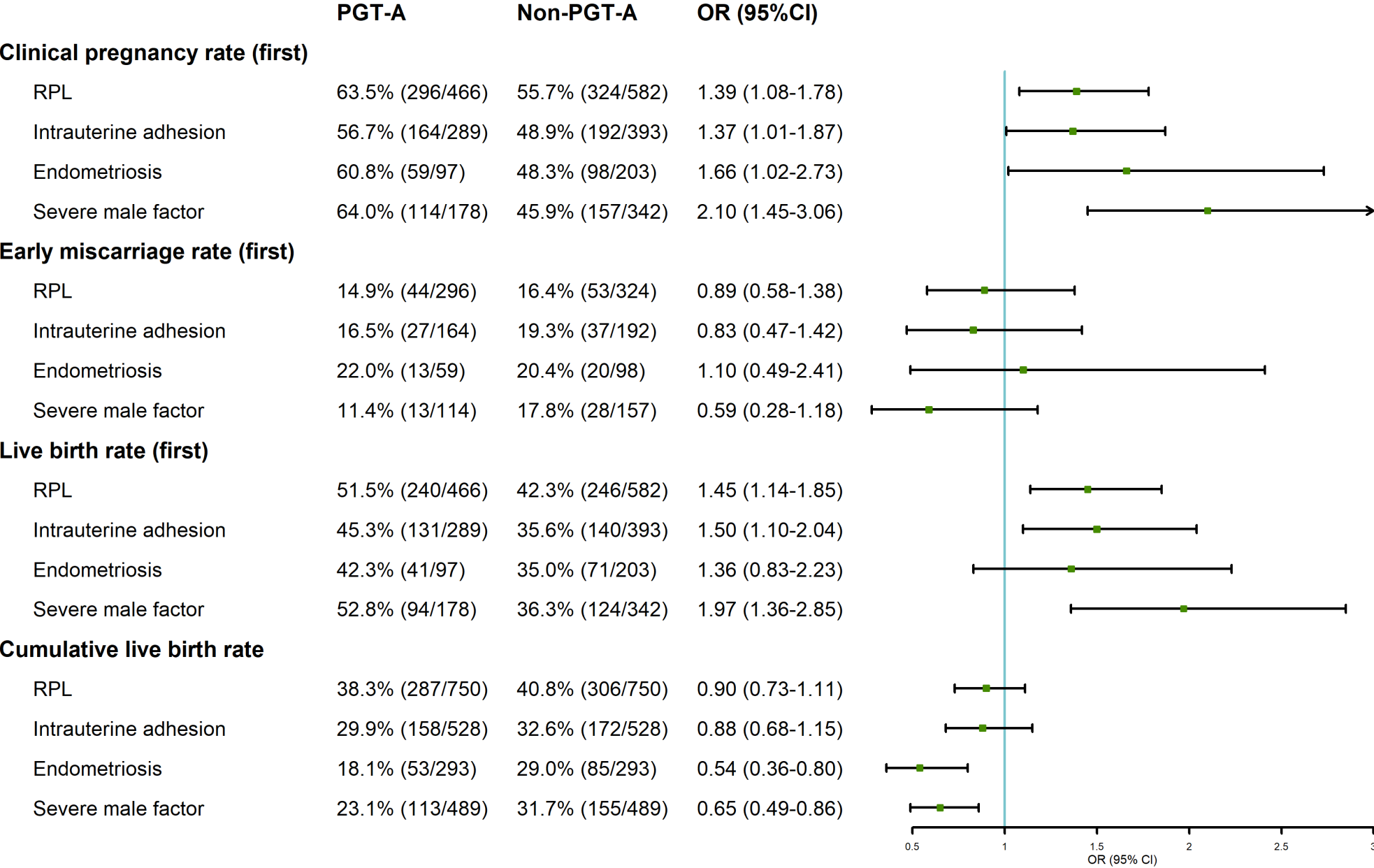

Supplement: Supplementary file 1 — Additional file 1: Figure S1. Distribution of distance among women who did (1) and did not (0) use preimplantation genetic testing for aneuploidy before and after the propensity score matching. Figure S2. Forest plot for sensitivity analyses of the primary outcomes between PGT-A and non-PGT-A groups. CI, confidence interval; OR, odds ratio; PGT-A, preimplantation genetic testing for aneuploidy. PSM, propensity score matching; PS stratification, propensity score stratification. Figure S3. Forest plot for female BMI-stratified comparison of the primary outcomes between PGT-A and non-PGT-A groups. BMI, body mass index; CI, confidence interval; OR, odds ratio; PGT-A, preimplantation genetic testing for aneuploidy. Figure S4. Forest plot for ovarian reserve and response-stratified comparison of the primary outcomes between PGT-A and non-PGT-A groups. AFC, antral follicle count; CI, confidence interval; OR, odds ratio; PGT-A, preimplantation genetic testing for aneuploidy. Figure S5. Forest plot for specific indication-stratified comparison of the primary outcomes between PGT-A and non-PGT-A groups. CI, confidence interval; OR, odds ratio; RPL, recurrent pregnancy loss; PGT-A, preimplantation genetic testing for aneuploidy. [file 12967_2023_4641_MOESM1_ESM.pdf]
