# Supplementary material for: Exploring the efficacy and beneficial population of preimplantation genetic testing for aneuploidy start from the oocyte retrieval cycle: a real-world study
Source: J Transl Med. 2023 Nov 2;21:779. doi: 10.1186/s12967-023-04641-2 (PMC10623718; doi:10.1186/s12967-023-04641-2)
Supplement: Supplementary file 2 — Additional file 2: Table S1. Female age-stratified outcomes of the first and cumulative transplant between PGT-A and non-PGT-A patients. Table S2. Stratified analyses of the first and cumulative transplant between PGT-A and non-PGT-A group by the presence or absence of RPL in women aged <35 years. Table S3. Female BMI-stratified outcomes of the first and cumulative transplant between PGT-A and non-PGT-A patients. Table S4. Ovarian reserve and response-stratified outcomes of the first and cumulative transplant between PGT-A and non-PGT patients. Table S5. Specific indication-stratified outcomes of the first and cumulative transplant between PGT-A and non-PGT-A patients. [file 12967_2023_4641_MOESM2_ESM.pdf]

Table S1. Female age-stratified outcomes of the first and cumulative transplant between PGT-A and non-PGT-A patients.

	Age<35y			Age 35-38y			Age 38-41y			Age 41-43y			Age≥43y		
	**PGT-A**	**Non-PGT-A**	**P value**	**PGT-A**	**Non-PGT-A**	**P value**	**PGT-A**	**Non-PGT-A**	**P value**	**PGT-A**	**Non-PGT-A**	**P value**	**PGT-A**	**Non-PGT-A**	**P value**
**Number of oocyte retrieval cycles**	817	817		672	672		1,025	1,025		612	612		755	755	
**Number of first transfer cycles**	657	720		419	558		441	783		128	391		41	369	
Single embryo transfer	98.9% (650/657)	19.9% (143/720)	<0.001	98.8% (414/419)	22.2% (124/558)	<0.001	98.6% (435/441)	20.9% (164/783)	<0.001	100% (128/128)	23.0% (90/391)	<0.001	100% (41/41)	25.7% (95/369)	<0.001
Live birth rate	54.3% (357/657)	54.7% (394/720)	0.886	51.6% (216/419)	47.0% (262/558)	0.155	50.1% (221/441)	27.5% (215/783)	<0.001	53.9% (69/128)	14.8% (58/391)	<0.001	46.3% (19/41)	5.1% (19/369)	<0.001
Singleton	53.3% (350/657)	38.6% (278/720)	<0.001	51.3% (215/419)	34.4% (192/558)	<0.001	49.7% (219/441)	24.8% (194/783)	<0.001	53.9% (69/128)	13.6% (53/391)	<0.001	43.9% (18/41)	4.9% (18/369)	<0.001
Multiple	1.1% (7/657)	16.1% (116/720)	<0.001	0.2% (1/419)	12.5% (70/558)	<0.001	0.5% (2/441)	2.7% (21/783)	0.006	0% (0/128)	1.3% (5/391)	0.31	2.4% (1/41)	0.3% (1/369)	0.119
Clinical pregnancy rate	65.0% (427/657)	67.1% (483/720)	0.413	62.8% (263/419)	60.2% (336/558)	0.417	61.5% (271/441)	44.8% (351/783)	<0.001	66.4% (85/128)	27.4% (107/391)	<0.001	63.4% (26/41)	13.6% (50/369)	<0.001
Singleton	62.9% (413/657)	44.6% (321/720)	<0.001	61.6% (258/419)	42.8% (239/558)	<0.001	60.5% (267/441)	38.1% (298/783)	<0.001	65.6% (84/128)	23.8% (93/391)	<0.001	61.0% (25/41)	12.7% (47/369)	<0.001
Multiple	2.1% (14/657)	22.5% (162/720)	<0.001	1.2% (5/419)	17.4% (97/558)	<0.001	0.9% (4/441)	6.8% (53/783)	<0.001	0.8% (1/128)	3.6% (14/391)	0.136	2.4% (1/41)	0.8% (3/369)	0.339
Pregnancy loss rate	16.4% (70/427)	18.4% (89/483)	0.42	17.9% (47/263)	22.0% (74/336)	0.21	18.5% (50/271)	38.7% (136/351)	<0.001	18.8% (16/85)	45.8% (49/107)	<0.001	26.9% (7/26)	62.0% (31/50)	0.004
Ectopic pregnancy	0.9% (4/427)	3.1% (15/483)	0.022	0.8% (2/263)	1.2% (4/336)	0.603	1.5% (4/271)	2.3% (8/351)	0.474	0% (0/85)	2.8% (3/107)	0.102	0% (0/26)	0% (0/50)	-
Early miscarriage	13.1% (56/427)	11.2% (54/483)	0.372	13.7% (36/263)	15.8% (53/336)	0.477	11.8% (32/271)	31.1% (109/351)	<0.001	11.8% (10/85)	42.1% (45/107)	<0.001	19.2% (5/26)	62.0% (31/50)	<0.001
Late miscarriage	2.3% (10/427)	4.1% (20/483)	0.134	3.8% (10/263)	5.4% (18/336)	0.373	5.2% (14/271)	5.4% (19/351)	0.892	7.1% (6/85)	1.9% (2/107)	0.096	7.7% (2/26)	0% (0/50)	0.11
Pregnancy complications	30.7% (131/427)	37.3% (180/483)	0.037	33.8% (89/263)	34.8% (117/336)	0.802	35.1% (95/271)	23.6% (83/351)	0.002	41.2% (35/85)	23.4% (25/107)	0.008	23.1% (6/26)	18.0% (9/50)	0.599
Gestational hypertension rate	2.8% (12/427)	4.6% (22/483)	0.17	4.9% (13/263)	2.7% (9/336)	0.15	4.8% (13/271)	1.4% (5/351)	0.019	7.1% (6/85)	2.8% (3/107)	0.18	3.8% (1/26)	4.0% (2/50)	0.974
Gestational diabetes rate	16.4% (70/427)	15.9% (77/483)	0.854	15.6% (41/263)	16.4% (55/336)	0.796	18.5% (50/271)	11.4% (40/351)	0.013	20.0% (17/85)	9.3% (10/107)	0.035	19.2% (5/26)	6.0% (3/50)	0.09
Preterm birth rate	9.8% (35/357)	20.9% (82/393)	<0.001	7.4% (16/216)	20.6% (54/262)	<0.001	8.6% (19/221)	13.5% (29/215)	0.105	7.2% (5/69)	10.3% (6/58)	0.538	26.3% (5/19)	21.1% (4/19)	0.903
Singleton	9.1% (32/350)	6.5% (18/277)	0.227	7.0% (15/215)	11.5% (22/192)	0.12	8.2% (18/219)	10.3% (20/194)	0.464	7.2% (5/69)	7.5% (4/53)	0.95	22.2% (4/18)	16.7% (3/18)	0.675
Multiple	42.9% (3/7)	55.2% (64/116)	0.529	100% (1/1)	45.7% (32/70)	0.991	50.0% (1/2)	42.9% (9/21)	0.846	-	40.0% (2/5)	-	100% (1/1)	100% (1/1)	0.967
Low birth weight rate	7.8% (28/361)	25.2% (128/508)	<0.001	2.8% (6/216)	19.0% (63/332)	<0.001	3.2% (7/218)	10.7% (25/233)	0.002	4.3% (3/69)	9.8% (6/61)	0.231	20.0% (4/20)	25.0% (5/20)	0.705
Singleton	6.3% (22/347)	4.7% (13/276)	0.382	2.3% (5/214)	4.7% (9/190)	0.197	2.8% (6/214)	4.2% (8/191)	0.449	4.3% (3/69)	3.9% (2/51)	0.908	11.1% (2/18)	16.7% (3/18)	0.632
Multiple	42.9% (6/14)	49.6% (115/232)	0.627	50.0% (1/2)	38.0% (54/142)	0.732	25.0% (1/4)	40.5% (17/42)	0.551	-	40.0% (4/10)	-	100% (2/2)	100% (2/2)	0.963
Neonatal malformation cycle rate	0.6% (2/357)	2.3% (9/394)	0.05	1.4% (3/216)	2.3% (6/262)	0.475	0.5% (1/221)	2.3% (5/215)	0.132	0% (0/69)	3.4% (2/58)	0.2	5.3% (1/19)	0% (0/19)	0.927
**Cumulative number of transfer cycles**	842	963		532	728		511	1,017		141	493		42	425	
Cumulative number of newborns	454	624		273	415		257	306		73	89		20	25	
Cumulative live birth rate[a]	54.2% (443/817)	58.5% (478/817)	0.081	39.9% (268/672)	49.3% (331/672)	<0.001	24.9% (255/1,025)	27.1% (278/1,025)	0.247	11.9% (73/612)	13.4% (82/612)	0.439	2.5% (19/755)	3.2% (24/755)	0.44
Singleton	53.2% (435/817)	41.5% (339/817)	<0.001	39.4% (265/672)	37.4% (251/672)	0.432	24.7% (253/1,025)	24.5% (251/1,025)	0.918	11.9% (73/612)	12.4% (76/612)	0.8793	2.4% (18/755)	3.0% (23/755)	0.43
Multiple	1.0% (8/817)	17.0% (139/817)	<0.001	0.4% (3/672)	11.9% (80/672)	<0.001	0.2% (2/1,025)	2.6% (27/1,025)	<0.001	0% (0/612)	1.0% (6/612)	0.031	0.1% (1/755)	0.1% (1/755)	0.997
Interval since oocyte retrievaled, days	408 (355-485)	265 (253-358)	<0.001	394 (345-485)	264 (253-352)	<0.001	378 (345-450)	266 (255-349)	<0.001	381 (339-441)	266 (255-366)	<0.001	338 (316-374)	264 (257-352)	0.007
**Number of cycles not reached live birth**	374	339	0.214	404	341		770	747		539	530		736	731	
Oocyte unretrieved cycle rate	0.1% (1/817)	0.2% (2/817)	0.571	1.9% (13/672)	2.5% (17/672)	0.461	5.9% (60/1,025)	4.7% (48/1,025)	0.236	8.3% (51/612)	9.3% (57/612)	0.546	14.7% (111/755)	14.4% (109/755)	0.88
No transferable embryo cycle rate[b]	11.0% (90/817)	8.0% (65/817)	0.035	24.4% (164/672)	12.4% (83/672)	<0.001	45.0% (461/1,025)	18.0% (185/1,025)	<0.001	66.2% (405/612)	29.7% (182/612)	<0.001	73.6% (556/755)	43.0% (325/755)	<0.001
With transferable embryo cycle rate[c]	20.7% (169/817)	19.1% (156/817)	0.421	16.4% (110/672)	16.1% (108/672)	0.882	8.6% (88/1,025)	19.2% (197/1,025)	<0.001	3.4% (21/612)	18.0% (110/612)	<0.001	0.9% (7/755)	16.0% (121/755)	<0.001

Notes: All ages were rounded down, the "left close, right open " rule of thumb was applied uniformly for stratification variables.

[a], twice or more live births under the same oocyte retrieval cycle are counted as one when the cumulative live birth rate is calculated. The numbers of twice live births in the PGT-A group and non-PGT-A group were 3 and 7 for women aged <35 years, 2 and 2 for women aged 35-38 years, 0 and 1 for women aged 38-41 years, 0 and 1 for women aged 41-43 years, 0 and 0 for women aged ⩾43 years, respectively;

[b], the proportions of abnormal embryos (including chimeras) detected in the PGT-A group of 5 age strata were 8.1% (66/817), 16.7% (112/672), 28.0% (287/1,025), 37.3% (228/612), 26.1% (197/755), respectively;

[c], the undetected transferable embryos were included in the PGT-A group;

Abbreviations: CI, confidence interval; Non-PGT-A, not use preimplantation genetic testing for aneuploidy; OR, odds ratio; PGT-A, preimplantation genetic testing for aneuploidy.

Table S2. Stratified analyses of the first and cumulative transplant between PGT-A and non-PGT-A group by the presence or absence of RPL in women aged <35 years .

	Age<35 without RPL			Age<35 with RPL		
	PGT-A	Non-PGT-A	P value	PGT-A	Non-PGT-A	P value
**Number of oocyte retrieval cycles**	320	320		387	387	
**Number of first transfer cycles**	255	299		301	333	
Single embryo transfer	98% (249/255)	16% (47/299)	<0.001	100% (309/310)	27% (89/333)	<0.001
Live birth rate	55.3% (141/255)	57.9% (173/299)	0.544	56.8% (176/310)	47.1% (157/333)	0.015
Singleton	54% (138/255)	39% (116/299)	<0.001	56% (174/310)	38% (125/333)	<0.001
Multiple	1% (3/255)	19% (57/299)	<0.001	1% (2/310)	10% (32/333)	<0.001
Clinical pregnancy rate	67.1% (171/255)	69.2% (207/299)	0.584	65.2% (202/310)	61.3% (204/333)	0.306
Singleton	65% (165/255)	43% (130/299)	<0.001	63% (196/310)	44% (146/333)	<0.001
Multiple	2% (6/255)	26% (77/299)	<0.001	2% (6/310)	17% (58/333)	<0.001
Pregnancy loss rate	18% (30/171)	16% (34/207)	0.773	13% (26/202)	23% (47/204)	0.008
Ectopic pregnancy	2% (3/171)	4% (8/207)	0.236	0% (1/202)	3% (7/204)	0.067
Early miscarriage	14.0% (24/171)	9.2% (19/207)	0.141	10.4% (21/202)	13.7% (28/204)	0.305
Late miscarriage	2% (3/171)	4% (9/207)	0.166	2% (4/202)	5% (11/204)	0.08
Pregnancy complications	32% (54/171)	31% (65/207)	0.97	29% (58/202)	37% (76/204)	0.068
Gestational hypertension rate	5% (9/171)	4% (9/207)	0.678	1% (2/202)	3% (7/204)	0.117
Gestational diabetes rate	16% (27/171)	16% (33/207)	0.968	15% (31/202)	15% (31/204)	0.966
Preterm birth rate	11% (15/141)	24% (41/173)	0.003	9% (15/176)	20% (32/157)	0.002
Singleton	10% (14/138)	9% (10/116)	0.679	8% (14/174)	6% (8/125)	0.592
Multiple	33% (1/3)	54% (31/57)	0.488	50% (1/2)	75% (24/32)	0.455
Low birth weight rate	9% (13/142)	27% (61/229)	<0.001	5% (9/178)	22% (42/188)	<0.001
Singleton	9% (12/136)	4% (5/115)	0.168	4% (7/174)	6% (8/124)	0.3
Multiple	17% (1/6)	49% (56/114)	0.157	50% (2/4)	53% (34/64)	0.903
Neonatal malformation cycle rate	1% (1/141)	2% (3/173)	0.436	1% (1/176)	1% (2/157)	0.508
**Cumulative number of transfer cycles**	320	398		394	458	
Cumulative number of newborns	183	291		211	243	
Cumulative live birth rate[a]	55.0% (176/320)	68.4% (219/320)	<0.001	54.0% (209/387)	51.7% (200/387)	0.517
Singleton	54% (172/320)	48% (152/320)	0.11	53% (207/387)	41% (158/387)	<0.001
Multiple	1% (4/320)	21% (67/320)	<0.001	1% (2/387)	11% (42/387)	<0.001
Interval since oocyte retrievaled, days	388 (350-479)	263 (252-354)	<0.001	404 (355-468)	272 (259-362)	<0.001
**Number of cycles not reached live birth**	144	101		178	187	
Oocyte unretrieved cycle rate	0% (1/320)	1% (3/320)	0.6	0% (0/387)	0% (1/387)	-
No transferable embryo cycle rate[b]	10% (33/320)	5% (17/320)	0.018	11% (43/387)	10% (39/387)	0.705
With transferable embryo cycle rate[c]	21.9% (70/320)	9.1% (29/320)	<0.001	20.9% (81/387)	22.2% (86/387)	0.657

Notes:

[a], twice or more live births under the same oocyte retrieval cycle are counted as one when the cumulative live birth rate is calculated. The numbers of twice live births in the PGT-A group and non-PGT-A group were 3 and 5 for women aged <35 years without RSA, 0 and 1 for women aged <35 years with RSA, respectively;

[b], the proportions of abnormal embryos (including chimeras) detected in the PGT-A group of 2 strata were 6.9% (22/320) and 8.5% (33/387), respectively;

[c], the undetected transferable embryos were included in the PGT-A group;

Abbreviations: CI, confidence interval; Non-PGT-A, not use preimplantation genetic testing for aneuploidy; OR, odds ratio; PGT-A, preimplantation genetic testing for aneuploidy; RPL, recurrent pregnancy loss.

Table S3. Female BMI-stratified outcomes of the first and cumulative transplant between PGT-A and non-PGT-A patients.

	BMI <18.5			BMI 18.5-24			BMI ≧24		
	PGT-A	Non-PGT-A	P value	PGT-A	Non-PGT-A	P value	PGT-A	Non-PGT-A	P value
**Number of oocyte retrieval cycles**	166	166		3,035	3,035		803	803	
**Number of first transfer cycles**	85	122		1,352	2,180		278	558	
Single embryo transfer	98.9% (84/85)	30.3% (37/122)	<0.001	99.0% (1,338/1,352)	22.7% (495/2,180)	<0.001	98.6% (274/278)	22.6% (126/558)	<0.001
Live birth rate	57.6% (49/85)	43.4% (53/122)	0.044	52.3% (707/1,352)	34.3% (748/2,180)	<0.001	51.1% (142/278)	32.4% (181/558)	<0.001
Singleton	56.5% (48/85)	31.1% (38/122)	<0.001	51.8% (701/1,352)	27.6% (601/2,180)	<0.001	49.6% (138/278)	26.2% (146/558)	<0.001
Multiple	1.2% (1/85)	12.3% (15/122)	0.003	0.4% (6/1,352)	6.7% (147/2,180)	<0.001	1.4% (4/278)	6.3% (35/558)	0.002
Clinical pregnancy rate	70.6% (60/85)	56.6% (69/122)	0.041	62.6% (847/1,352)	46.9% (1,023/2,180)	<0.001	65.8% (183/278)	43.7% (244/558)	<0.001
Singleton	69.4% (59/85)	37.7% (46/122)	<0.001	61.5% (831/1,352)	35.4% (771/2,180)	<0.001	63.3% (176/278)	33.5% (187/558)	<0.001
Multiple	1.2% (1/85)	18.9% (23/122)	<0.001	1.2% (16/1,352)	11.6% (252/2,180)	<0.001	2.5% (7/278)	10.2% (57/558)	<0.001
Pregnancy loss rate	18.3% (11/60)	23.2% (16/69)	0.5	16.5% (140/847)	26.9% (275/1,023)	<0.001	22.4% (41/183)	25.8% (63/244)	0.416
Ectopic pregnancy	1.7% (1/60)	1.4% (1/69)	0.921	1.1% (9/847)	2.4% (25/1,023)	0.026	0% (0/183)	0.8% (2/244)	0.5
Early miscarriage	13.3% (8/60)	21.7% (15/69)	0.217	11.8% (100/847)	19.6% (201/1,023)	<0.001	17.5% (32/183)	21.3% (52/244)	0.326
Late miscarriage	3.3% (2/60)	0% (0/69)	0.21	3.7% (31/847)	4.8% (49/1,023)	0.231	4.9% (9/183)	3.7% (9/244)	0.533
Pregnancy complications	25.0% (15/60)	30.4% (21/69)	0.493	33.8% (286/847)	30.8% (315/1,023)	0.17	37.7% (69/183)	32.0% (78/244)	0.217
Gestational hypertension rate	3.3% (2/60)	1.4% (1/69)	0.491	3.3% (28/847)	2.4% (25/1,023)	0.265	8.7% (16/183)	6.1% (15/244)	0.309
Gestational diabetes rate	11.7% (7/60)	13.0% (9/69)	0.813	18.2% (154/847)	14.4% (147/1,023)	0.026	19.1% (35/183)	15.2% (37/244)	0.28
Preterm birth rate	4.1% (2/49)	15.1% (8/53)	0.095	8.2% (58/707)	17.3% (129/747)	<0.001	12.0% (17/142)	23.2% (42/181)	0.011
Singleton	4.2% (2/48)	5.3% (2/38)	0.911	8.0% (56/701)	7.7% (46/601)	0.823	9.4% (13/138)	12.3% (18/146)	0.433
Multiple	0% (0/1)	40.0% (6/15)	0.997	33% (2/6)	57% (83/146)	0.272	100% (4/4)	68.6% (24/35)	0.3
Low birth weight rate	2.1% (1/48)	25.0% (17/68)	<0.001	5.1% (36/708)	19.8% (175/886)	<0.001	6.2% (9/145)	16.4% (35/213)	0.004
Singleton	2% (1/46)	13% (5/38)	0.087	4.3% (30/696)	5.2% (31/595)	0.448	3.6% (5/137)	4.8% (7/145)	0.625
Multiple	0% (0/2)	40.0% (12/30)	0.5	50.0% (6/12)	49.5% (144/291)	0.972	50.0% (4/8)	41.2% (28/68)	0.634
Neonatal malformation cycle rate	0% (0/49)	0% (0/53)	-	0.7% (5/707)	1.7% (13/748)	0.075	2.8% (4/142)	2.2% (4/181)	0.728
**Cumulative number of transfer cycles**	101	150		1,803	2,660		333	706	
Cumulative number of newborns	56	78		866	1,131		173	272	
Cumulative live birth rate[a]	33.1% (55/166)	38.0% (63/166)	0.359	28.0% (851/3,035)	30.9% (938/3,035)	0.014	21.0% (169/803)	28.5% (229/803)	<0.001
Singleton	32.5% (54/166)	28.9% (48/166)	0.476	27.7% (842/3,035)	24.8% (754/3,035)	0.01	20.5% (165/803)	23.2% (186/803)	0.205
Multiple	0.6% (1/166)	9.0% (15/166)	<0.001	0.3% (9/3,035)	6.1% (184/3,035)	<0.001	0.5% (4/803)	5.4% (43/803)	<0.001
Interval since oocyte retrievaled, days	391 (355-459)	261 (254-372)	<0.001	395 (348-474)	264 (255-352)	<0.001	393 (349-464)	262 (251-335)	<0.001
**Number of cycles not reached live birth**	111	103		2,184	2,097		634	574	
Oocyte unretrieved cycle rate	7.2% (12/166)	3.6% (6/166)	0.154	5.3% (162/3,035)	6.5% (197/3,035)	0.057	9.2% (74/803)	8.0% (64/803)	0.374
No transferable embryo cycle rate[b]	34.3% (57/166)	24.1% (40/166)	0.041	42.1% (1,278/3,035)	22.6 (687/3,035)	<0.001	51.3% (412/803)	24.9% (200/803)	<0.001
With transferable embryo cycle rate[c]	13.9% (23/166)	13.9% (23/166)	0.988	10.0% (304/3,035)	18.0% (545/3,035)	<0.001	8.5% (68/803)	15.4% (124/803)	<0.001

Notes:

[a], twice or more live births under the same oocyte retrieval cycle are counted as one when the cumulative live birth rate is calculated. The numbers of twice live births in the PGT-A group and non-PGT-A group were 0 and 0 for BMI <18.5 strata, 6 and 8 for women BMI 18.5-23.9 strata, 0 and 0 for BMI over 24 strata, respectively;

[b], the proportions of abnormal embryos (including chimeras) detected in the PGT-A group of 3 strata were 19.9% (33/166), 22.9% (696/3,035) and 25% (203/803), respectively;

[c], the undetected transferable embryos were included in the PGT-A group;

Abbreviations: BMI, body mass index; CI, confidence interval; Non-PGT-A, not use preimplantation genetic testing for aneuploidy; OR, odds ratio; PGT-A, preimplantation genetic testing for aneuploidy.

Table S4. Ovarian reserve and response-stratified outcomes of the first and cumulative transplant between PGT-A and non-PGT-

	AFC<5			AFC≥5 & Recovered oocyte ≤9			AFC≥5 & Recovered oocyte ＞9		
	PGT-A	Non-PGT-A	P value	PGT-A	Non-PGT-A	P value	PGT-A	Non-PGT-A	P value
**Number of oocyte retrieval cycles**	710	710		1,854	1,854		1,338	1,338	
**Number of first transfer cycles**	73	264		603	1,323		1,003	1,267	
Single embryo transfer	100% (73/73)	41.7% (110/264)	<0.001	98.8% (596/603)	20.6% (273/1,323)	<0.001	98.8% (991/1,003)	18.7% (237/1,267)	<0.001
Live birth rate	39.7% (29/73)	15.9% (42/264)	<0.001	48.9% (295/603)	27.5% (364/1,323)	<0.001	55.4% (556/1,003)	44.8% (567/1,267)	<0.001
Singleton	39.7% (29/73)	14.8% (39/264)	<0.001	48.8% (294/603)	23.7% (314/1,323)	<0.001	54.4% (546/1,003)	33.5% (425/1,267)	<0.001
Multiple	0% (0/73)	1.1% (3/264)	0.994	0.2% (1/603)	3.8% (50/1,323)	<0.001	1.0% (10/1,003)	11.2% (142/1,267)	<0.001
Clinical pregnancy rate	53.4% (39/73)	26.1% (69/264)	<0.001	60.4% (364/603)	38.9% (515/1,323)	<0.001	66.9% (671/1,003)	58.9% (746/1,267)	<0.001
Singleton	52.1% (38/73)	23.9% (63/264)	<0.001	59.0% (356/603)	31.0% (410/1,323)	<0.001	65.5% (657/1,003)	40.0% (507/1,267)	<0.001
Multiple	1.4% (1/73)	2.3% (6/264)	0.636	1.3% (8/603)	7.9% (105/1,323)	<0.001	1.4% (14/1,003)	18.9% (239/1,267)	<0.001
Pregnancy loss rate	25.6% (10/39)	39.1% (27/69)	0.159	19.0% (69/364)	29.3% (151/515)	<0.001	17.1% (115/671)	24.0% (179/746)	0.001
Ectopic pregnancy	2.6% (1/39)	1.4% (1/69)	0.684	0.8% (3/364)	2.5% (13/515)	0.078	0.9% (6/671)	2.3% (17/746)	0.039
Early miscarriage	23.1% (9/39)	34.8% (24/69)	0.207	13.7% (50/364)	22.3% (115/515)	0.001	12.4% (83/671)	16.9% (126/746)	0.017
Late miscarriage	0% (0/39)	2.9% (2/69)	0.5	4.4% (16/364)	4.5% (23/515)	0.96	4.0% (27/671)	4.7% (35/746)	0.54
Pregnancy complications	12.8% (5/39)	24.6% (17/69)	0.14	36.3% (132/364)	30.7% (158/515)	0.083	32.2% (216/671)	30.6% (228/746)	0.51
Gestational hypertension rate	0% (0/39)	2.9% (2/69)	0.5	5.8% (21/364)	3.5% (18/515)	0.11	3.7% (25/671)	3.4% (25/746)	0.703
Gestational diabetes rate	7.7% (3/39)	14.5% (10/69)	0.305	16.8% (61/364)	14.4% (74/515)	0.334	17.1% (115/671)	11.3% (84/746)	0.001
Preterm birth rate	13.8% (4/29)	9.5% (4/42)	0.578	8.1% (24/295)	13.2% (48/364)	0.039	9.2% (51/556)	20.7% (117/564)	<0.001
Singleton	13.8% (4/29)	10.3% (4/39)	0.655	7.8% (23/294)	7.0% (22/314)	0.701	8.4% (46/546)	9.0% (38/423)	0.759
Multiple	-	0% (0/3)	-	100% (1/1)	52.0% (26/50)	0.992	50.0% (5/10)	56.0% (79/141)	0.711
Low birth weight rate	3.6% (1/28)	6.8% (3/44)	0.564	4.4% (13/294)	16.2% (66/408)	<0.001	5.5% (31/560)	20.9% (146/699)	<0.001
Singleton	3.6% (1/28)	7.9% (3/38)	0.478	4.5% (13/292)	5.2% (16/310)	0.685	3.9% (21/540)	5.0% (21/419)	0.4
Multiple	-	0% (0/6)	-	0% (0/2)	51.0% (50/98)	0.5	50.0% (10/20)	44.6% (125/280)	0.642
Neonatal malformation cycle rate	0% (0/29)	0% (0/42)	-	1.4% (4/295)	1.4% (5/364)	0.984	0.9% (5/556)	2.3% (13/567)	0.063
**Cumulative number of transfer cycles**	79	281		691	1,576		1,281	1,775	
Cumulative number of newborns	31	49		330	477		705	935	
Cumulative live birth rate[a]	4.4% (31/710)	6.5% (46/710)	0.081	17.6% (326/1,854)	22.5% (418/1,854)	<0.001	51.5% (689/1,338)	56.7% (758/1,338)	0.007
Singleton	4.4% (31/710)	6.1% (43/710)	0.154	17.5% (325/1,854)	19.5% (362/1,854)	0.118	50.5% (676/1,338)	43.6% (584/1,338)	<0.001
Multiple	0% (0/710)	0.4% (3/710)	0.2	0.1% (1/1,854)	3.0% (56/1,854)	<0.001	1.0% (13/1,338)	13.0% (174/1,338)	<0.001
Interval since oocyte retrievaled, days	373 (337-427)	264 (256-304)	<0.001	382 (346-449)	261 (254-319)	<0.001	401 (349-483)	268 (255-362)	<0.001
**Number of cycles not reached live birth**	679	664		1,528	1,436		649	580	
Oocyte unretrieved cycle rate	22.1% (157/710)	21.8% (155/710)	0.898	4.4% (81/1,854)	4.2% (78/1,854)	0.808	0% (0/1,338)	0% (0/1,338)	-
No transferable embryo cycle rate[b]	67.5% (479/710)	52.3% (371/710)	<0.001	53.8% (998/1,854)	23.3% (432/1,854)	<0.001	16.8% (225/1,338)	3.3% (44/1,338)	<0.001
With transferable embryo cycle rate[c]	2.4% (17/710)	12.8% (91/710)	<0.001	7.3% (136/1,854)	14.3% (266/1,854)	<0.001	17.3% (231/1,338)	22.9% (306/1,338)	<0.001

Notes:

[a], twice or more live births under the same oocyte retrieval cycle are counted as one when the cumulative live birth rate is calculated. The numbers of twice live births in the PGT-A group and non-PGT-A group were 0 and 0 for women with AFC<5, 3 and 3 for women with AFC≧ 5 & Recovered oocyte≦9, 3 and 3 for women with AFC ≧5 & Recovered oocyte >9, respectively;

[b], the proportions of abnormal embryos (including chimeras) detected in the PGT-A group of 3 strata were 21.0% (149/710), 30.5% (566/1,854) and 14.1% (189/1,338), respectively;

[c], the undetected transferable embryos were included in the PGT-A group;

Abbreviations: AFC, antral follicle count; CI, confidence interval; Non-PGT-A, not use preimplantation genetic testing for aneuploidy; OR, odds ratio; PGT-A, preimplantation genetic testing for

Table S5. Specific indication-stratified outcomes of the first and cumulative transplant between PGT-A and non-PGT-A patients.

	RPL			Intrauterine adhesion			Endometriosis			Severe male factor		
	**PGT-A**	**Non-PGT-A**	**P value**	**PGT-A**	**Non-PGT-A**	**P value**	**PGT-A**	**Non-PGT-A**	**P value**	**PGT-A**	**Non-PGT-A**	**P value**
**Number of oocyte retrieval cycles**	750	750		528	528		293	293		489	489	
**Number of first transfer cycles**	466	582		289	393		97	203		178	342	
Single embryo transfer	99.6% (464/466)	24.9% (145/582)	<0.001	99.7% (288/289)	31.0% (122/393)	<0.001	99.0% (96/97)	20.7% (42/203)	<0.001	100% (178/178)	20.8% (71/342)	<0.001
Live birth rate	51.5% (240/466)	42.3% (246/582)	0.003	45.3% (131/289)	35.6% (140/393)	0.01	42.3% (41/97)	35.0% (71/203)	0.223	52.8% (94/178)	36.3% (124/342)	<0.001
Singleton	50.9% (237/466)	33.7% (196/582)	<0.001	44.6% (129/289)	29.8% (117/393)	<0.001	42.3% (41/97)	28.6% (58/203)	0.018	52.8% (94/178)	30.7% (105/342)	<0.001
Multiple	0.6% (3/466)	8.6% (50/582)	<0.001	0.7% (2/289)	5.9% (23/393)	<0.001	0% (0/97)	6.4% (13/203)	0.011	0% (0/178)	5.6% (19/342)	0.001
Clinical pregnancy rate	63.5% (296/466)	55.7% (324/582)	0.01	56.7% (164/289)	48.9% (192/393)	0.041	60.8% (59/97)	48.3% (98/203)	0.042	64.0% (114/178)	45.9% (157/342)	<0.001
Singleton	61.6% (287/466)	39.3% (229/582)	<0.001	55.0% (159/289)	38.2% (150/393)	<0.001	58.8% (57/97)	36.0% (73/203)	<0.001	632.9 (112/178)	36.0% (123/342)	<0.001
Multiple	1.9% (9/466)	16.3% (95/582)	<0.001	1.7% (5/289)	10.7% (42/393)	<0.001	2.1% (2/97)	12.3% (25/203)	0.004	1.1% (2/178)	9.9% (34/342)	<0.001
Pregnancy loss rate	18.9% (56/296)	24.1% (78/324)	0.12	20.1% (33/164)	27.1% (52/192)	0.126	30.5% (18/59)	27.6% (27/98)	0.692	17.5% (20/114)	21.0% (33/157)	0.477
Ectopic pregnancy	0.3% (1/296)	1.9% (6/324)	0.113	0% (0/164)	2.6% (5/192)	0.065	1.7% (1/59)	3.1% (3/98)	0.604	0.9% (1/114)	0% (0/157)	0.4
Early miscarriage	14.9% (44/296)	16.4% (53/324)	0.609	16.5% (27/164)	19.3% (37/192)	0.492	22.0% (13/59)	20.4% (20/98)	0.809	11.4% (13/114)	17.8% (28/157)	0.148
Late miscarriage	3.7% (11/296)	5.6% (18/324)	0.282	3.7% (6/164)	6.2% (12/192)	0.271	6.8% (4/59)	5.1% (5/98)	0.662	5.3% (6/114)	3.2% (5/157)	0.397
Pregnancy complications	31.1% (92/296)	36.7% (119/324)	0.139	32.9% (54/164)	39.6% (76/192)	0.194	25.4% (15/59)	28.6% (28/98)	0.669	28.1% (32/114)	30.6% (48/157)	0.656
Gestational hypertension rate	2.0% (6/296)	3.7% (12/324)	0.221	4.3% (7/164)	3.1% (6/192)	0.568	1.7% (1/59)	3.1% (3/98)	0.604	4.4% (5/114)	3.2% (5/157)	0.606
Gestational diabetes rate	17.2% (51/296)	17.0% (55/324)	0.933	17.1% (28/164)	20.8% (40/192)	0.369	18.6% (11/59)	9.2% (9/98)	0.085	18.4% (21/114)	12.1% (19/157)	0.15
Preterm birth rate	9.2% (22/240)	20.7% (51/246)	<0.001	6.9% (9/131)	15.7% (22/140)	0.022	7.3% (3/41)	15.5% (11/71)	0.218	4.3% (4/94)	10.6% (13/123)	0.086
Singleton	8.4% (20/237)	8.2% (16/196)	0.918	6.2% (8/129)	6.8% (8/117)	0.84	7.3% (3/41)	10.3% (6/58)	0.607	4.3% (4/94)	7.7% (8/104)	0.318
Multiple	66.7% (2/3)	70.0% (35/50)	0.903	50.0% (1/2)	60.9% (14/23)	0.765	-	38.5% (5/13)	-	-	26.3% (5/19)	-
Low birth weight rate	4.5% (11/242)	21.3% (63/296)	<0.001	7% (9/131)	14% (23/162)	0.046	5.0% (2/40)	10.8% (9/83)	0.299	3.2% (3/93)	14.8% (21/142)	0.004
Singleton	3.4% (8/236)	5.6% (11/196)	0.267	4.7% (6/127)	4.3% (5/116)	0.877	5.0% (2/40)	3.5% (2/57)	0.718	3.2% (3/93)	5.8% (6/104)	0.5
Multiple	50.0% (3/6)	52.0% (52/100)	0.924	75.0% (3/4)	39.1% (18/46)	0.197	-	26.9% (7/26)	-	-	39.5% (15/38)	-
Neonatal malformation cycle rate	0.4% (1/240)	2.0% (5/246)	0.145	0% (0/131)	3.6% (5/140)	0.061	2.4% (1/41)	2.8% (2/71)	0.905	1.1% (1/94)	1.6% (2/124)	0.732
**Cumulative number of transfer cycles**	592	774		364	509		124	253		212	422	
Cumulative number of newborns	291	373		160	204		54	101		114	179	
Cumulative live birth rate[a]	38.3% (287/750)	40.8% (306/750)	0.316	29.9% (158/528)	32.6% (172/528)	0.353	18.1% (53/293)	29.0% (85/293)	0.002	23.1% (113/489)	31.7% (155/489)	0.003
Singleton	37.9% (284/750)	32.1% (241/750)	0.02	29.5% (156/528)	26.7% (141/528)	0.305	18.1% (53/293)	23.5% (69/293)	0.104	22.9% (112/489)	27.0% (132/489)	0.14
Multiple	0.4% (3/750)	8.7% (65/750)	<0.001	0.4% (2/528)	5.9% (31/528)	<0.001	0% (0/293)	5.5% (16/293)	<0.001	0.2% (1/489)	4.7% (23/489)	<0.001
Interval since oocyte retrievaled, days	420 (362-492)	272 (257-378)	<0.001	424 (377-484)	324 (260-375)	<0.001	428 (392-534)	260 (252-320)	<0.001	380 (346-488)	261 (254-330)	<0.001
**Number of cycles not reached live birth**	463	444		370	356		240	208		376	334	
Oocyte unretrieved cycle rate	2.1% (16/750)	3.5% (26/750)	0.121	3.6% (19/528)	3.8% (20/528)	0.87	9.9% (29/293)	6.1% (18/293)	0.094	7.8% (38/489)	8.6% (42/489)	0.6
No transferable embryo cycle rate[b]	26.3% (197/750)	16.4% (123/750)	<0.001	33.3% (176/528)	18.2% (96/528)	<0.001	47.1% (138/293)	22.9% (67/293)	<0.001	45.6% (223/489)	25.4% (124/489)	<0.001
With transferable embryo cycle rate[c]	16.5% (124/750)	22.3% (167/750)	0.005	14.6% (77/528)	25.6% (135/528)	<0.001	8.2% (24/293)	19.8% (58/293)	<0.001	7.2% (35/489)	12.7% (62/489)	0.004

Notes:

[a], twice or more live births under the same oocyte retrieval cycle are counted as one when the cumulative live birth rate is calculated. The numbers of twice live births in the PGT-A group and non-PGT-A group were 1 and 2 for women with RSA, 0 and 1 for women with intrauterine adhesion, 0 and 1 for women with endometriosis, 0 and 1 for couples with severe male factor, respectively;

[b], the proportions of abnormal embryos (including chimeras) detected in the PGT-A group of 4 strata were 18.3% (137/750), 19.5% (103/528), 27.3% (80/293) and 24.7% (121/489), respectively;

[c], the undetected transferable embryos were included in the PGT-A group;

Abbreviations: CI, confidence interval; Non-PGT-A, not use preimplantation genetic testing for aneuploidy; OR, odds ratio; PGT-A, preimplantation genetic testing for aneuploidy; RPL, recurrent pregnancy loss.
